# Dual enhancement in the radiosensitivity of prostate cancer through nanoparticles and chemotherapeutics

**DOI:** 10.1186/s12645-023-00228-0

**Published:** 2023-09-29

**Authors:** Nolan Jackson, Iona Hill, Abdulaziz Alhussan, Kyle Bromma, Jessica Morgan, Belal Abousaida, Yasmin Zahra, Yuri Mackeyev, Wayne Beckham, Steven Herchko, Sunil Krishnan, Devika Basnagge Chithrani

**Affiliations:** 1 Department of Physics and Astronomy, University of Victoria, Victoria, BC V8P 5C2 Canada; 2 Vivian L. Smith Department of Neurosurgery, The University of Texas Health Science Center, Houston, TX 77030 USA; 3 Department of Biochemistry and Microbiology, University of Victoria, Victoria, BC V8P 5C2 Canada; 4 Trev and Joyce Deeley Research Centre, BC Cancer, Victoria, BC V8R 6V5 Canada; 5 Department of Radiation Oncology, Mayo Clinic, Jacksonville, FL 32224 USA; 6 British Columbia Cancer-Victoria, Victoria, BC V8R 6V5 Canada; 7 Centre for Advanced Materials and Related Technologies, Department of Chemistry, University of Victoria, Victoria, BC V8P 5C2 Canada; 8 Division of Medical Sciences, University of Victoria, Victoria, BC V8P 5C2 Canada; 9 Department of Computer Science, Mathematics, Physics and Statistics, Okanagan Campus, University of British Columbia, Kelowna, BC V1V 1V7 Canada

**Keywords:** Gold nanoparticles, Docetaxel, Prostate cancer, Radiotherapy, Radiosensitivity

## Abstract

**Background:**

Radiotherapy (RT) is an essential component in the treatment regimens for many cancer patients. However, the dose escalation required to improve curative results is hindered due to the normal tissue toxicity that is induced. The introduction of radiosensitizers to RT treatment is an avenue that is currently being explored to overcome this issue. By introducing radiosensitizers into tumor sites, it is possible to preferentially enhance the local dose deposited. Gold nanoparticles (GNPs) are a potential candidate that have shown great promise in increasing the radiosensitivity of cancer cells through an enhancement in DNA damage. Furthermore, docetaxel (DTX) is a chemotherapeutic agent that arrests cells in the G2/M phase of the cell cycle, the phase most sensitive to radiation damage. We hypothesized that by incorporating DTX to GNP-enhanced radiotherapy treatment, we could further improve the radiosensitization experienced by cancer cells. To assess this strategy, we analyzed the radiotherapeutic effects on monolayer cell cultures in vitro, as well as on a mice prostate xenograft model in vivo while using clinically feasible concentrations for both GNPs and DTX.

**Results:**

The introduction of DTX to GNP-enhanced radiotherapy further increased the radiotherapeutic effects experienced by cancer cells. A 38% increase in DNA double-strand breaks was observed with the combination of GNP/DTX vs GNP alone after a dose of 2 Gy was administered. In vivo results displayed significant reduction in tumor growth over a 30-day observation period with the treatment of GNP/DTX/RT when compared to GNP/RT after a single 5 Gy dose was given to mice. The treatment strategy also resulted in 100% mice survival, which was not observed for other treatment conditions.

**Conclusions:**

Incorporating DTX to work in unison with GNPs and RT can increase the efficacy of RT treatment. Our study suggests that the treatment strategy could improve tumor control through local dose enhancement. As the concentrations used in this study are clinically feasible, there is potential for this strategy to be translated into clinical settings.

**Supplementary Information:**

The online version contains supplementary material available at 10.1186/s12645-023-00228-0.

## Introduction

Although advancements have been made in improving the treatment of cancer, it is still one the leading causes of death in North America. Statistics show that approximately 40% of North Americans will develop cancer in their lifetime, and 1 out of 5 will succumb to the disease (S.C.a.t.P.H.A.o.C 2021; Cronin et al. 2018; Siegel, et al. 2022). The most common form of treatment to combat cancer is radiotherapy (RT), alongside chemotherapy and surgery. Integrating the systemic effects of chemotherapy with targeted local RT treatments has been shown to greatly increase the cure rates of solid tumors (Rubin and Carter 1976; Yapp et al. 1997). By further enhancing localized RT, there is tremendous potential to maximize the effect of dose deposited to tumors while still maintaining low levels of normal tissue toxicity. One of the current strategies to preferentially increase treatment efficacy is to incorporate a radiosensitizer to work in unison with RT.

The use of gold nanoparticles (GNPs) as radiosensitizers has gained a lot of interest over the past decade. This concept of using high atomic number (*Z*) materials as radiosensitizers was first introduced using iodine (*Z*_I_ = 53) as a radiosensitizer. Early studies showed that iodine could increase the radiosensitization of cells both in vitro and in vivo (Santos Mello et al. 1983; Matsudaira et al. 1980). However, among other materials, employing GNPs as a radiosensitizing agent has attracted more attention due to their high atomic number (*Z*_Au_ = 79), biocompatibility, higher circulation lifetime, and the possibility of tumor targeting (Hainfeld et al. 2004; Schuemann et al. 2016). Previous studies have shown radiosensitizing properties of GNPs using keV and clinically relevant MeV energy photons (Chithrani et al. 2010; Tudda et al. 2022; Zhao et al. 2019). This radiosensitization due to GNPs is attributed to increased photoelectric photon absorption, particularly at kilovoltage photon energies, by high-Z materials compared with soft tissue. The addition of GNPs results in a shower of secondary electrons that can increase the output of DNA damaging free radicals. Based on Monte Carlo calculations, the predicted radiation dose enhancement due to kilovoltage photon energies is much larger than megavoltage photon energies (Cho 2005). However, the extent of GNP-enhanced sensitization observed at clinically relevant MeV energies is significantly higher than the predicted values by Monte Carlo studies (Chithrani et al. 2010). The exact mechanism by which sensitization occurs at clinically relevant MeV photons remains unclear, but it may be physical, chemical or biological. One potential radiosensitizing mechanism could be attributed to the oxidative stress induced due to GNPs. GNPs can catalyze chemical reactions to produce reactive oxygen species (ROS). Due to the electron active surface of GNPs, they have the ability to produce ROS via electron transfer to molecular oxygen, where the introduction of irradiation could augment this effect (Chen et al. 2020). Furthermore, it has been suggested that GNPs bind to thiol-containing endogenous reducing agents of cells, such as glutathione and N-acetyl cysteine; thus decreasing the antioxidant levels (Korman et al. 2021; Her et al. 2017). However, the precise underlying mechanisms that contribute to the radiosensitization and the extent at which they contribute still remains to be fully understood, and further research is required to elucidate the exact mechanisms behind the observed radiosensitization and their contribution at clinically relevant MeV energies. Nevertheless, incorporating GNPs into RT treatment could have substantial impacts in the treatment of cancer.

One of the current strategies used in the clinic to treat cancer is the use of chemotherapeutic drugs, such as docetaxel (DTX), paclitaxel, gemcitabine, and cisplatin. Although radiation is not generally used concomitantly with these systemic drugs, the combination of chemotherapeutics and RT could greatly improve curative results. Recent clinical trials have shown better survival in prostate cancer patients as well as head and neck cancer patients when RT was combined with a lower dose of DTX (Kumar 2003; Kumar et al. 2003; Karasawa et al. 2006; Kim and Khuri 2002). In phase I/II trials, the weekly DTX dose was reduced from 25 to 40 mg/m^2^ to 20 mg/m^2^ for prostate cancer and to 10 mg/m^2^ in locally advanced head and neck cancer (Kumar 2003; Kumar et al. 2003; Karasawa et al. 2006). DTX has ideal properties as a radiosensitizer, since its mechanism of action has forms of cytotoxicity that are complementary to the lethal effects of radiation. DTX binds to free tubulin and promotes the assembly of tubulin into stable microtubules, preventing tumor cell division, and, therefore, arresting cell population in the G2/M phase of the cell cycle (Paoletti et al. 1997; Granger et al. 2014). On the other hand, ionizing radiation causes direct and indirect DNA structural damage, especially during the G2/M phase of the cell cycle, which disrupts viable cell division, eventually leading to tumor cell death. Considering this synergy between radiation and DTX, the combined use of DTX/RT has become an attractive therapeutic strategy.

**Fig. 1:**
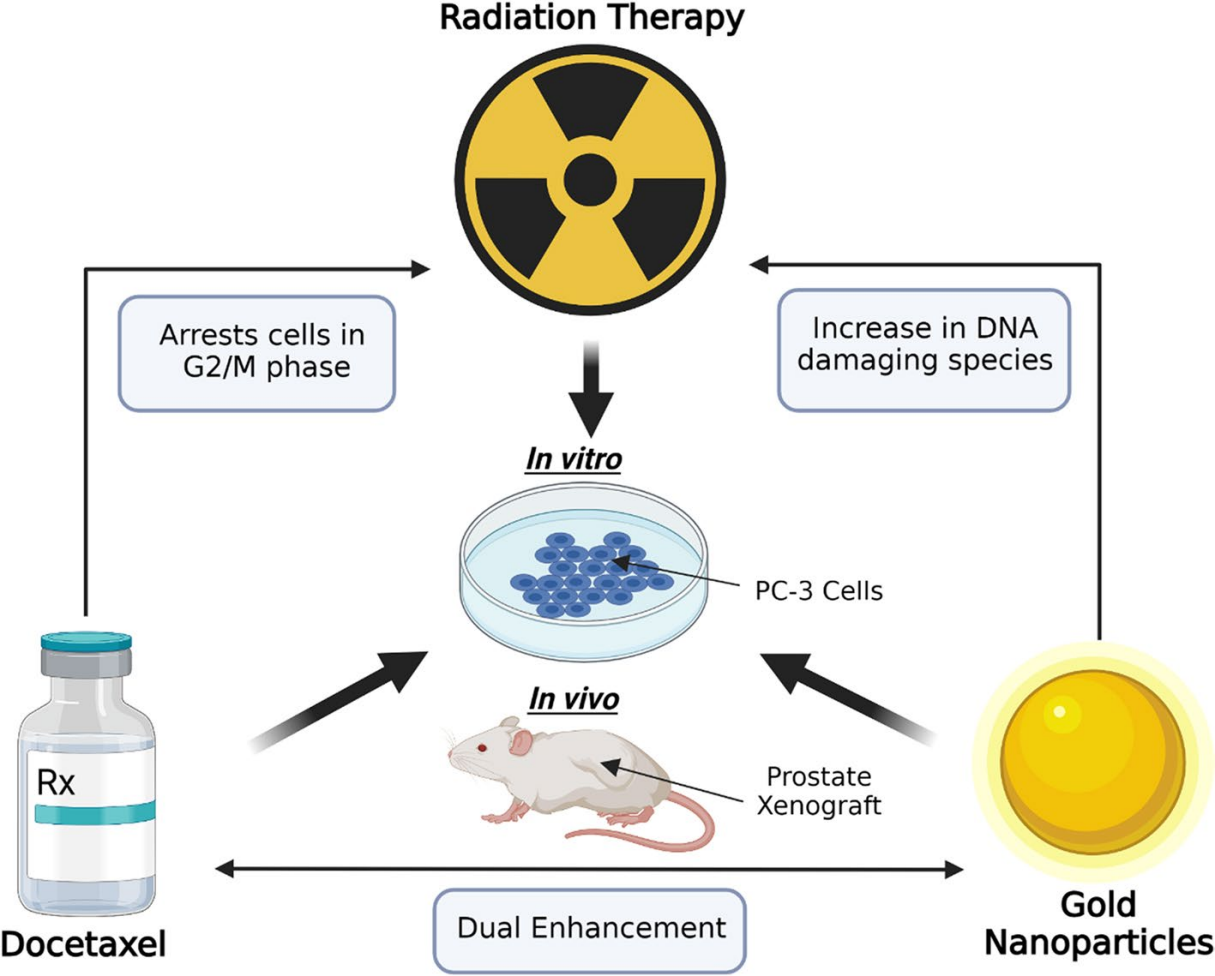
Schematic figure outlining the strategy of the triple combination of gold nanoparticles (GNPs), docetaxel (DTX), and radiotherapy (RT). Both GNPs and DTX sensitize cells to radiation, amplifying the dose delivered, which can result in the enhancement of radiotherapeutic damage induced to tumor cells. Efficacy of treatment strategy was examined on both in vitro and in vivo prostate models. (Created using Biorender.com)

The goal of this current study is to investigate the combined effect of GNPs, DTX and RT with megavoltage radiation both in vitro and in vivo, as shown in Fig. 1*.* The efficacy of radiotherapy as a form of treatment is constrained due to the normal tissue toxicity, imposing dose limits that directly contribute to undesirable treatment outcomes. It is our belief that incorporating GNPs and DTX into RT treatment could enhance the radiotherapeutic damage to tumor cells, and, therefore, increase the tumor control and curative results. However, a potential secondary application of the treatment strategy is to achieve similar tumor control to standard radiotherapy treatment using a de-escalated dose regimen of both DTX and RT for an optimized therapeutic result, where side effects associated with RT and chemotherapy could be minimized. GNPs are biocompatible and have shown very promising radiosensitization effects both in vitro and in vivo (Chithrani et al. 2010; Wolfe et al. 2015). DTX concurrent to RT, has also been well-tolerated in phase 1–2 trials (Perrotti et al. 2008; Guttilla et al. 2014; Carles et al. 2019). The use of GNPs in combination with DTX as a therapeutic strategy for the treatment of cancer has also been pursued by other research groups. Although, due to indiscriminate distribution throughout the body of systemic DTX, most of the in vitro studies have fixated on the use of GNPs as a delivery vehicle for DTX, with the concentration of GNPs used being in the micromolar range (Thambiraj et al. 2021; Thambiraj et al. 2019; Gotov et al. 2018). For our in vitro studies, we used GNPs as a radiosensitizing agent at nanomolar concentrations.

A recent study by Guo et al. went one step further and demonstrated how GNPs can be used to deliver DTX in vivo to enhance the radiosensitivity of prostate xenograft tumor models (Guo et al. 2021). In their study, mice were treated with DTX loaded GNPs and received a radiation dose of 6 Gy using a 6 MeV electron beam. An injected dose of GNPs of 5 mg/kg was used and a significant reduction in tumor growth after irradiation was seen over a 14-day observation period. However, in our study, we used an injected dose of GNPs of 2 mg/kg and monitored the tumor growth for 30 days after irradiation with clinical 6 MV X-rays. Furthermore, we showed 100% survival for GNPs/DTX/RT treated mice up to 50-day post-treatment, and it is a significant improvement compared to other treatment options. Furthermore, GNPs have been tested in Phase I/II clinical trials (Zhang et al. 2023) and free DTX is currently tested with RT in the clinic for high-risk prostate cancer patients (Perrotti et al. 2008; Guttilla et al. 2014; Carles et al. 2019). Therefore, GNPs in combination with free DTX is more feasible to accelerate into clinical trials. The overall aim of this study is to develop a therapeutic strategy to enhance tumor sensitivity to radiation and/or chemotherapy for curative purposes.

## Materials and methods

### Gold nanoparticle synthesis, functionalization, and characterization

A citrate reduction method was used to synthesize GNPs with a core diameter of ~ 11 nm. After adding 28.82 mL of ddH_2_O to an Erlenmeyer flask, 1.18 mL of 1% tetrachloroauric acid (HAuCl_4_) solution was added to the flask. This was done with the flask being heated on a hot plate, while the solution was vigorously stirred. After the solution began boiling, 1.2 mL of 5% tribasic sodium citrate was rapidly added to the solution. The color of solution changed to ruby red, which indicates the successful synthesis of GNPs. The solution was then left to boil for another 5 min while being stirred. It was then removed from the heat and brought to room temperature.

The GNPs used in this study were functionalized using polyethylene glycol thiol (PEG), MW of 2000 Da, and a peptide containing the integrin-binding domain RGD (CKKKKKKGG**RGD**MFG), MW of 1600 Da. The cysteine residue of the RGD peptide enables conjugation with the GNPs via a thiol bond. PEG was added to the colloidal GNP solution, such that there was 1 molecule of PEG per nm^2^ of GNP surface area. The resulting solution was then stirred to allow for conjugation. RGD was then added to the GNP + PEG solution at an optimized ratio of 2:1 PEG molecules to RGD peptide molecules. After RGD peptide was added to solution, the solution was then stirred to achieve successful conjugation to form the GNP + PEG/RGD complex. For 11 nm GNPs, this resulted in a theoretical determination of 380 PEG molecules and 190 RGD molecules per GNP. However, physical measurements of ligand packing density on GNPs were not performed.

Characterizations of the GNP, GNP + PEG, and GNP + PEG/RGD colloidal solutions were performed using ultraviolet–visible (UV–Vis) spectrometry (Perkin Elmer λ 365 Spectrophotometer), dynamic light scattering (DLS) and ζ potential analysis (Anton Paar LiteSizer 500) methods. UV–Vis spectrometry was performed on the solutions to estimate the size and concentration of GNPs. The hydrodynamic diameter and surface charge of the GNPs were measured by DLS and ζ potential measurements, respectively. Scanning Transmission Electron Microscopy (STEM) (Ultra-high Resolution STEM SU9000, Hitachi) was used to verify the shape and size of GNPs.

### Cell and culture conditions

Human Prostate cancer cell line, PC-3 (ATCC#: CRL-1435^™^), was purchased from the American Type Culture Centre (ATCC). For in vitro experiments, cells were cultured using high glucose Dulbecco’s Modified Eagle medium (DMEM; Gibco) that was supplemented with 4 mM GlutaMax (Gibco), 10% Fetal Bovine Serum (Gibco) and 1% penicillin/streptomycin (Gibco). Washing of cell cultures was performed using phosphate-buffered saline (PBS), and TrypLE (Gibco) was used for cell detachment. For in vivo experiments, cells were cultured in F12 cell culture medium (ATCC) supplemented with 10% (v/v) foetal bovine serum (FBS) and 100 μg/mL penicillin streptomycin (Gibco). Cells were incubated at 37 °C with 5% CO_2_ and routinely sub cultured once 70–80% confluency was reached.

### Live cell imaging

Cells were plated on 35 mm coverslip bottom dishes (MatTek, Ashland, MA USA). Following a 24 h incubation, cells cultures were dosed at a concentration of 7.5 µg/mL of functionalized Cy5-labeled GNP complex, as well as DTX at a concentration of 2.72 nM. Cell cultures were then incubated for 24 h. NucBlue^™^ Live ReadyProbes^™^ Reagent (R37605; ThermoFisher Scientific, Waltham, MA, USA) containing Hoechst 33,342 dye and CellLight^™^ Tubulin–GFP BacMam 2.0 (ThermoFisher Scientific, Waltham, MA, USA) was used to stain nuclei and microtubules, respectively, prior to imaging. Images were taken using a 60 × oil immersion objective lens using a confocal laser scanning microscope (Zeiss LSM 980, Carl ZeissMicroscopy GmbH, Jena, Germany).

### Analysis of gold nanoparticle uptake and retention

For the in vitro experiment, cells were plated in 6-well dishes and incubated for 24 h. Cell cultures were then dosed with GNP, DTX, or GNP/DTX and incubated for another 24 h period. The concentrations of GNPs and DTX cells were treated with was 7.5 µg/mL and 2.72 nM, respectively. Once cells were ready for processing, they were washed 3 times using PBS and were dissociated using TrypLE (Gibco). Fresh media was added and mixed in each well, and 1 mL of sample was transferred into Eppendorf tubes. A hemocytometer was then used to count the number of cells present in 1 mL of sample. For the in vivo experiment, each weighed tissue sample was placed into 2 mL of TrypLE and blended for further disassociation using a handheld homogenizer (Fisher). For both in vitro and in vivo experiments, 500 µL of each sample was transferred into glass tubes and treated with 250 µL of aqua regia (3:1 molar ratio of HCl and HNO_3_ (VWR)). Samples were placed into a 90 °C mineral oil bath and heated for 30 min. 100 µL of hydrogen peroxide was then added to each sample and samples remained in the oil bath for another 30 min. Finally, deionized water was used to dilute each sample to a 2.5% v/v (volume per volume) acid content. To measure the gold content in each sample, inductively coupled plasma-mass spectrometry (ICP-MS; Agilent 8800 Triple Quadrupole, Agilent Technologies, Santa Clara, CA, USA) was used by comparing with gold standard solutions prepared from 1000 mg/ml stock solutions (Agilent) by serial dilution. The number of gold nanoparticles present in each cell was calculated using the following:

$$\frac{\frac{Gold\,Conc\mathrm{entration}}{Sample}\left[\frac{g}{mL}\right]\times Sample\,Vol\mathrm{ume }\left[mL\right]\times {N}_{A}[\frac{atoms}{mol}]}{Gold\,atomic\,mass\left[\frac{g}{mol}\right]\times \mathrm{Number }\,of\,Cells\times \frac{ Gold\,atoms}{Gold\,nanoparticle}}$$

where *N*_*A*_ is Avogadro's number. The number gold atoms per GNP is calculated by the following equation:

$$\frac{Atoms\,per\,unit\,cell\times Gold\,Nanoparticle\,Vol\mathrm{ume }\left[n{m}^{3}\right]}{Unit\,cell\,Vol\mathrm{ume }\left[n{m}^{3}\right]}=\frac{4\times \frac{4\pi {r}^{3}}{3}}{{a}^{3}}=\frac{2}{3}\pi {\left(\frac{D}{a}\right)}^{3}$$

where *a* = 0.408 nm, the length of a unit cell, and *D* is the core diameter of the spherical GNPs. The GNPs were synthesized such that it results in a face-centered cubic lattice with 4 atoms per unit cell. For the calculations, it was assumed that there is homogeneity in the size of GNPs.

### DNA damage assay/dark-field hyper spectral imaging (HSI)

Cells were seeded in 6-well dishes that contained glass coverslips and were incubated for 24 h. Following the incubation period, cell cultures were then dosed with either GNP, DTX, GNP/DTX, or left untreated for control samples. The concentration of GNP and DTX used in this assay was 7.5 µg/mL and 2.72 nM, respectively. Cell cultures were then incubated for another 24 h before a radiation dose of 2 Gy was administered. Post-irradiation, media in each well was removed and was replaced with fresh media before cells were incubated. 24 h after irradiation, cells were washed twice using PBS before they were fixed with 4% paraformaldehyde (PFA). To assess the DNA double-strand breaks in cells, a DNA repair protein, 53BP1, was fluorescently labelled. This was accomplished using labeled antibodies against the repair protein. Cells were first dosed with 2% BSA/0.1% Triton-X in PBS and incubated for 20 min. The primary antibody solution was then diluted 1:200 using 0.5% BSA/0.1% Triton-X/PBS. The diluted solution was then placed on top of parafilm, and glass coverslips were placed face down onto the solution. Samples were left in a dark room to incubate for 1 h. Post-incubation, glass coverslips were washed twice with PBS, and once with 0.5% BSA/0.1% Triton-X/PBS. A secondary antibody solution was diluted 1:500 with 0.5% BSA/0.1% Triton-X/PBS and placed onto parafilm. Coverslips were placed face down onto solution and left to incubate for 30 min in a dark room at room temperature. Coverslips were again washed twice with PBS and mounted onto slides with ProLong^™^ (P36930; ThermoFisherScientific, Waltham, MA, USA) Glass Antifade Mountant for imaging. 53BP1 foci were imaged using a 60 × oil immersion lens using a confocal laser scanning microscope (Zeiss LSM 980). The secondary antibody used to fluorescently label the 53BP1 protein was tagged with Alex Fluor 488 (excitation 490 nm, emission 525 nm). 53BP1 images were processed and foci and nuclei were counted. Hyper spectral images were taken with a dark-field microscope to visualize the GNP accumulation and cell morphology. This was accomplished using a 60 × oil immersion objective lens and a hyperspectral camera (CytoViva, Auburn, AL, USA).

### Radiation proliferation assay

Cells were equally seeded into 96-well plates and incubated for 24 h before being dosed with either GNP, DTX, GNP/DTX or left untreated for control samples, at concentrations of 7.5 µg/mL and 2.72 nM of GNPs and DTX, respectively. After initial treatment, cells were incubated for another 24 h. Following irradiation of cells at a dose of 5 Gy, media was removed from each well and replaced with fresh media. A 10% solution of PrestoBlue^™^ (A13261; ThermoFisher, Waltham, MA, USA) in media was added to wells in preparation of measurements. Cells were then incubated for 1.5 h before measurements were taken. Multiple samples were prepared, such that a new sample was measured for each timepoint. Measurements of cell viability were performed using a CytationOne^™^ Multi-Reader (filters at excitation of 530/25 nm and emission of 590/35 nm, Winooski, VT, USA).

### Cell cycle analysis

For in vitro cell cycle analysis, cells were plated in 6-well dishes. Following 24 h of incubation, cells were dosed with either GNP, DTX, or GNP/DTX. One well was left un-dosed for the control sample. Samples were processed 24 h post-dosing. For cell processing, cells were dissociated using TrypLE (Gibco) and resuspended in media before being transferred into 15 mL falcon tubes. For the in vivo analysis, samples were placed in a solution of Collagenase/Dispase (Roche 10269638001; Sigma Aldrich, St. Louis, MO, USA) for 2 h before being filtered through a 100 µm cell strainer. Both in vitro and in vivo samples were then processed according to the following steps. Cells were centrifuged at 300xg for 5 min at 4 °C before being washed with PBS. This was repeated twice. The cell pellet was resuspended in 1% PFA in PBS, added dropwise while vortexing, and incubated in the dark on ice for 15 min for cell fixation. Cells were rewashed and centrifuged again. Cells were suspended in 0.6 mL of PBS and 1.4 mL of freezer cold 100% ethanol and placed into a −20 °C freezer until further processing.

Samples were centrifuged at 350xg for 10 min at 20 °C to create a cell pellet. Following centrifugation, the pellets were suspended in 1 mL of 0.5% BSA in PBS and centrifuged again for 5 min at 350xg at 20 °C. Samples were resuspended in 0.5% BSA/0.1% Triton-X 100, in PBS preceded by RNaseA at a concentration of 100 µg/mL. Samples were then shaken for 25 min at 37 °C. Propidium iodide (excitation: 488 nm; emission: 600 nm) was added to the samples at a concentration of 10 µg/mL, and samples were shaken for 1 h at 4 °C. The samples were centrifuged again at 350xg for 5 min at 20 °C before being resuspended in 1 mL of 1% BSA/PBS and filtered through a 50 µm cell strainer. Samples were then analyzed using Flow Cytometry (BD FACS Calibur).

### In vitro cellular irradiation

All in vitro radiation assays were done with a clinical 6 MV linear accelerator (Varian Truebeam, Palo Alto, CA, USA), and samples were placed between two 30 cm × 30 cm × 5 cm solid water (Gammex-RMI, Middleton WI, USA) blocks. Samples were irradiated at a source-axis distance of 100 cm. A field size of 28 cm x 28 cm and a dose rate of 600 monitor units (MUs) per minute was used for all irradiations. The appropriate number of MUs to administer was calculated using the known calibration output of the machine. Non-irradiated samples were transported along with irradiated samples to maintain consistency.

### Preparation and treatment of xenograft

Athymic nude mice (NU/J, Jackson Laboratory) were used to evaluate the biodistribution of GNP/DTX and radiosensitization in vivo. All procedures involving animals were done according to IACUC-approved protocols (IACUC protocol No. A00004897). Mice were injected with 1 × 10^6^ PC-3 cells subcutaneously in the upper right thigh. Tumors in all mice were allowed to reach 7–8 mm in largest longitudinal diameter (length) before the mice were randomised into five treatment groups: (i) no treatment (control), (ii) RT alone, (iii) GNP/RT, (iv) DTX/RT, (v) GNP/DTX/RT. The mice were then treated with intravenously administered GNP, DTX, GNP/DTX, or no treatment.

### Biodistribution and cell cycle sample collection

Mice were prepared as described in “Preparation and Treatment of Xenograft” Section. Mice were sacrificed at either 24 h or 72 h timepoints after nanoparticle injection. The organs of the mice were harvested, including tumor, blood, skin, liver, kidney, spleen, heart, lung, brain and intestines. Prior to collection of the samples, clean glass vials were weighed. The dissected organ samples were added to the vials and the organs were lyophilised at 30 mTorr pressure for 48 h. Once the tissues were dried, the vials containing the organ samples were weighed, and the weight of the dried tissue then calculated. In addition, the long term biodistribution of the GNP was assessed by ICP-MS at the end point of the experiment (day 52 following randomisation). Three mice from each of the GNP, GNP/DTX, GNP/RT and GNP/DTX/RT treatment groups were sacrificed at the experimental end point, and the tumor, liver, kidney and spleen were harvested for ICP-MS analysis following the same protocol as previously described. For cell cycle sample collection, a section of tumor was fixed by submerging in 70% EtOH at room temperature.

### In vivo radiation study

Mice were prepared as described in “Preparation and Treatment of Xenograft” Section. Nine mice were used in each condition cohort. 24 h after injection of PBS (control), GNP,DTX, or GNP/DTX, the mice were treated with 5 Gy radiation. Mice were anesthetised with ketamine:xylazine (100 mg/kg:10 mg/kg), via intraperitoneal injection, 15 min before irradiation. Radiation was delivered using 6 MV photons on a Varian TrueBeam linear accelerator using a dose rate of 600 monitor units (MU) per minute. A 10 × 10 cm square field was set, and the tumor-bearing right thigh of a mouse was placed at one of the four corners of the radiation field with the collimator jaw shielding out all intraabdominal contents to reduce radiation enteritis. A piece of 1 cm bolus was placed on the surface of the tumor without any air gaps to bring the maximum dose to the desired depth, and a source to surface distance (SSD) of 100 cm was set to the top of the bolus. The number of MUs delivered was calculated using the desired dose and known calibration output of the TrueBeam machine. The tumor for each mouse was measured thrice weekly following radiation treatment. To estimate the tumor volume, the greatest longitudinal diameter (length) and greatest transversal diameter (width) were measured by vernier callipers, while the mice were conscious. The volume of the tumor was calculated by the modified ellipsoidal formula: *V* = ½ (Length x Width^2^). Mice were scarified once the longitudinal diameter of the tumor became greater than 18 mm, once mobility was lost or if tumor ulceration occurred.

### Statistical analysis

Statistical analysis was performed using the two-sample *t* test via the python package statannot (O.2.3). For statistical significance represented in figures, ns indicates no significance, * indicates 0.01 < *p* < 0.05, ** indicates 0.001 < *p* < 0.01, and *** indicates *p* < 0.001. All experiments besides the mouse study were repeated three times and the data presented are the average of all experiments. Error bars signify one standard deviation from the mean of the three independent measurements.

## Results and discussion

### Characterization of GNPs

Once GNPs are injected intravenously, they are expected to accumulate within the tumor by exploiting the leaky vasculature of tumors. As a result, it is important to functionalize GNPs to enhance the residence time in blood for their accumulation within the tumor. For this reason, the GNPs used in this study were functionalized with polyethylene glycol (PEG) as well as a peptide molecule containing the integrin-binding domain RGD. Based on previous studies, PEG molecules on GNP surfaces could significantly shield their uptake by macrophages, enabling them to bypass the immune system resulting in the enhancement of their residence time in blood (Suk et al. 2016). In our in vitro study, we incorporated PEG onto our GNP system considering its translation into in vivo studies followed by clinical studies. However, previous studies have also demonstrated lower uptake of GNPs with the incorporation of PEG (Cruje and Chithrani 2015). This can be rectified with the addition of targeting molecules, such as RGD peptides. RGD peptides bind preferentially to the αvβ3 integrin, which is overexpressed by many cancer cells, and this mechanism has been exploited by various targeted applications in cancer therapeutics and diagnostics (Danhier et al. 2012). Studies have also shown the conjugation with RGD facilitates a significant improvement in GNP uptake at single cell level, tissue level, and in vivo (Cruje et al. 2015; Yang et al. 2018). Since the αvβ3 integrin has been observed on PC-3 cells, we chose to use RGD as our targeting moiety to enhance the uptake of GNPs (Taylor et al. 2012; Sun et al. 2007; Stachurska et al. 2012). However, for successful conjugation with the RGD peptide, it requires a stabilizing molecule. PEG provides this stability, and thus plays a dual role in the functionalization process. It is also important to consider the size of the GNPs, PEG and RGD for optimizing the cellular uptake of GNPs. It has been previously shown that among the size range of 10–100 nm, GNPs with a diameter of 50 nm had the greatest cellular uptake at monolayer level (Chithrani et al. 2006). With the introduction of PEG and RGD, smaller GNPs have a higher surface curvature that allows for better receptor–ligand interaction at the cell membrane (Cruje et al. 2015). Therefore, we have chosen GNPs with a core diameter of ~ 11 nm for this study among the size range from 10 to 100 nm.

**Fig. 2:**
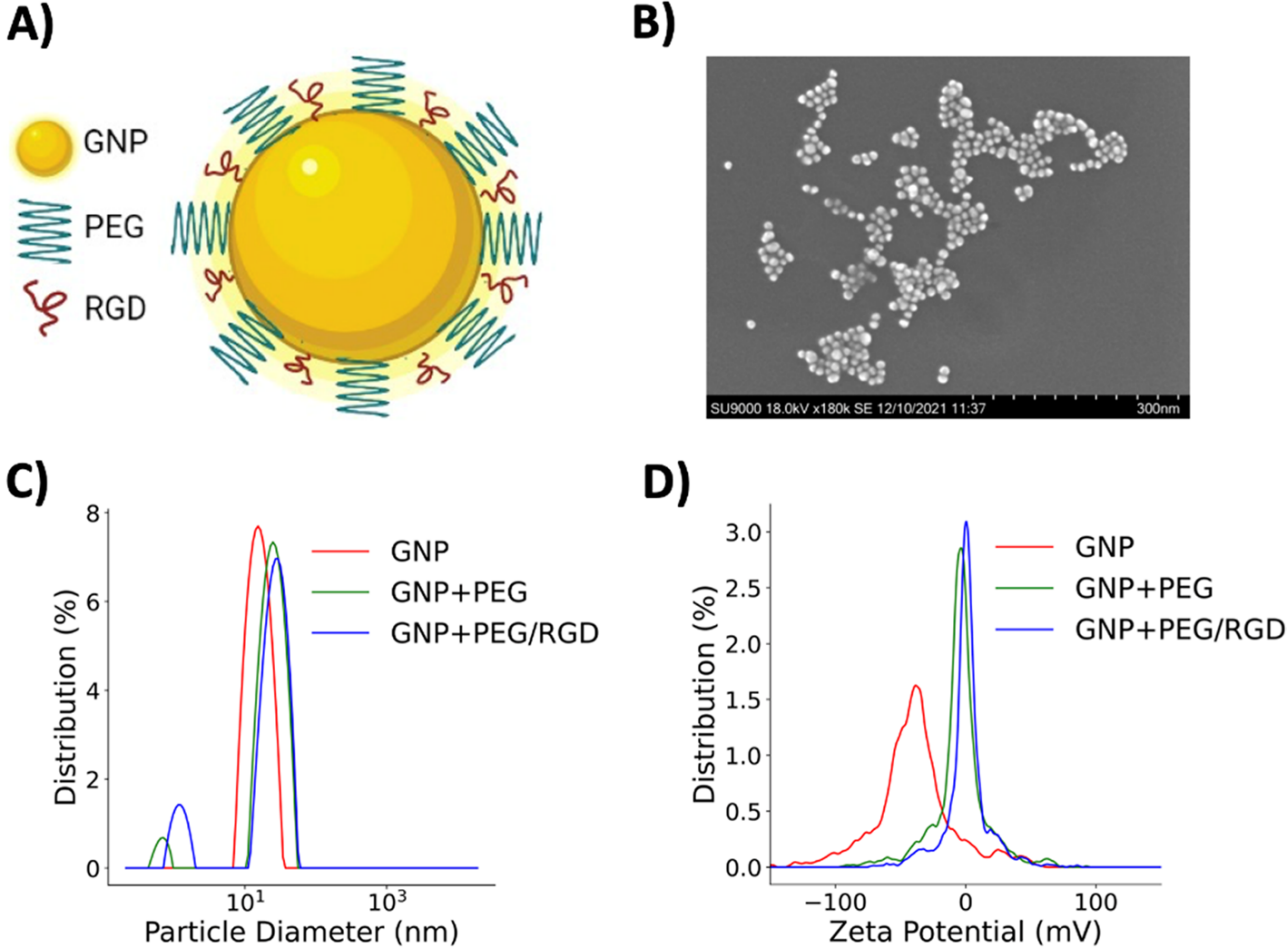
Characterization of GNPs. **A** Schematic diagram of strategic functionalization of gold nanoparticles (GNP) with polyethylene glycol (PEG) and RGD peptide. **B** Transmission electron microscopy (TEM) image of functionalized GNPs. **C**, **D** Measurements of the hydrodynamic diameter and zeta potential of GNPs at each step of the functionalization process

Various characterization measurements of the GNPs were performed using transmission electron microscopy (TEM), ultraviolet–visible spectrometry (UV–Vis), dynamic light scattering (DLS), and zeta potential analysis. According to the TEM images of as-made (citrate-capped) GNPs, the core diameter was ~ 11.32 nm ± 2.1 nm (Fig. 2B). UV–Vis, DLS, and zeta potential measurements were used to characterize the GNPs at each step of the functionalization process. GNPs were first functionalized with PEG, and then RGD molecules, via a sequential addition of PEG and RGD at optimized ratios of 2:1 PEG to RGD. Based on UV–Vis and DLS data, the addition of PEG and RGD peptides (molecular weights are 2000 and 1600 Da, respectively) resulted in an increase in the diameter of the GNPs. UV–Vis spectra peaks red shifted from 519 to 521 nm following conjugation with PEG and RGD peptide (Additional file 1: Figure S1A). Similarly, DLS measurements displayed that the hydrodynamic diameter increased at each step of the functionalization process. The citrate-capped GNPs had a hydrodynamic diameter of 16.9 ± 0.4 nm and increased to 26.6 ± 0.4 nm with the addition of PEG molecules. The hydrodynamic diameter further increased to 29.2 ± 0.5 nm following conjugation with RGD peptide (Fig. 2B; Additional file 1: Figure S1C). Measurements of the zeta potential were also performed at each step, as this technique is a good indicator to verify conjugation with PEG and RGD peptide. Due to the replacement of negatively charged citrate molecules with neutral PEG and positively charged RGD peptide on the GNP surface, the surface charge of the GNPs increased. The measured zeta potential of the citrate-stabilized GNPs was −39.2 ± 2.8 mV and increased to −1.1 ± 0.5 mV following the conjugation of PEG and RGD peptide (Fig. 2C; Additional file 1: Figure S1C). The data collected at each step of the functionalization process is summarized in the table in Additional file 1: Figure S1C. Additional DLS measurements of the GNP + PEG/RGD colloidal solution diluted in PBS and DMEM cell culture media were also performed to confirm the stability of the GNP complex Additional file 1: Figure S1B. For simplicity, GNP + PEG/RGD complex is referred to as GNPs or GNP from now onwards, and they were used for all forthcoming experiments.

### GNP and DTX enhancement of radiosensitization in vitro

**Fig. 3:**
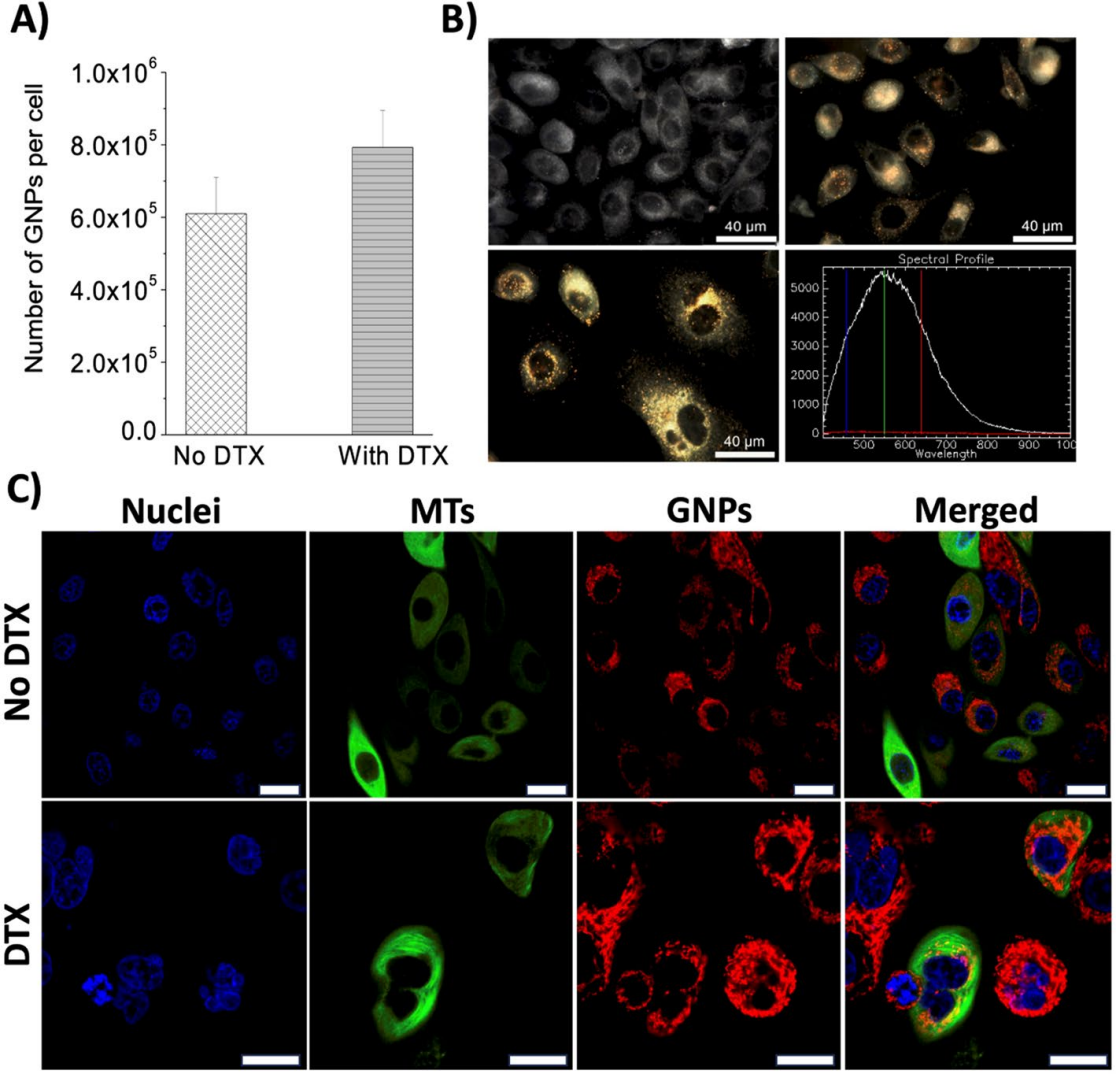
Cellular uptake and transportation of GNPs. **A** Quantitative analysis of GNP accumulation in PC-3 cells in the presence or absence of docetaxel (DTX). **B** Hyperspectral images of GNP accumulation in PC-3 cells and spectra collected from GNPs. Top image corresponds to control cells (no DTX), bottom image represents cells treated with DTX. **C** Live cell confocal images of intracellular distribution of GNPs in control cells (top row) and DTX treated cells (bottom row). Nuclei stained in blue (first column), microtubules stained in green (second column), GNPs stained in red (third column). All columns merged in fourth column. Scale bar is 20 µm

Our first goal was to establish the effect of DTX on the GNP uptake and transport process at the cellular level before evaluating the radiosensitization due to both agents. GNP cellular uptake is achieved via receptor mediated endocytosis (Chithrani 2010). Size and surface properties of GNPs play a vital role in their intracellular uptake and transportation (Chithrani and Chan 2007). Once GNPs enter the cell via endocytosis, they become trapped in endosomes followed by fusing with lysosomes for processing. Processed GNPs are then excreted from the cell via exocytosis. For this study, PC-3 cells were dosed at a concentration of ~ 1 nM (7.5 µg/mL) of GNPs, since such concentrations can be produced in vivo as well (Bromma et al. 2022). Cells were also treated simultaneously with DTX at a concentration of 2.72 nM, the IC-50, and incubated for 24 h before cell harvesting (Bromma et al. 2022). To quantify GNP uptake, inductively coupled surface plasma mass spectrometry (ICP-MS) was used. The results of our ICP-MS analysis are displayed in Fig. 3A. There was an insignificant difference between the accumulation of GNPs in control cells (untreated) vs DTX treated cells 24 h post-dosing. To further analyze the cellular uptake, we performed hyperspectral imaging on cell cultures to visualize the intracellular distribution of GNPs.

As shown in Fig. 3B, there appears to be a higher presence of gold within cells treated with DTX vs control cells. In the hyperspectral image of DTX treated cells, multinucleated cells can also be seen due to stabilized microtubules (MTs), which could be one of reasons for the increase in the accumulation of GNPs. As previously mentioned, DTX causes the formation of faulty MTs. Since MTs facilitate the transportation of GNPs within cells, the secretion of GNPs is hindered. The damaged MTs also prevents the proper formation of mitotic spindles required for cell division, thus the lower number of GNPs in the control cell population could also be attributed to the dilution of GNPs during cell division (Additional file 1: Section S2) (Kim et al. 2011; Bannister et al. 2020). Furthermore, with the treatment of DTX, there is a higher fraction of cell population in G2/M phase (Additional file 1: Section S4), and these cells are expected to have higher number of GNPs compared to other phases, since the cell has more time to accumulate GNPs before cell division. Figure 3C also displays live cell images of the accumulation of GNPs in control and DTX treated cells. As shown, DTX treated cells appear to have a greater accumulation of GNPs. Although our quantitative analysis of GNP accumulation in cells displayed no significant increase between control and DTX treated cells, our qualitative data suggest that DTX treated cells had a greater accumulation of GNPs. However, this effect of DTX on the cellular uptake of GNPs can also increase with an escalation in dose of DTX delivered to cells. Another study found that there was a twofold increase in the uptake of GNPs in PC-3 cells treated with 50 nM of DTX vs untreated cells (Alhussan et al. 2021). Even though endocytosis is not fully affected by MT stabilizing effect, exocytosis is very much affected, since it hinders transport of processed NPs to the cell periphery for their removal from the cells. This leads to trapping of GNPs within cells if the effect of DTX is present. Thus, not only does DTX sensitize cells to RT, but it also allows for greater accumulation of GNPs within tumor cells, producing a more effective therapeutic pathway when combined with RT.

**Fig. 4:**
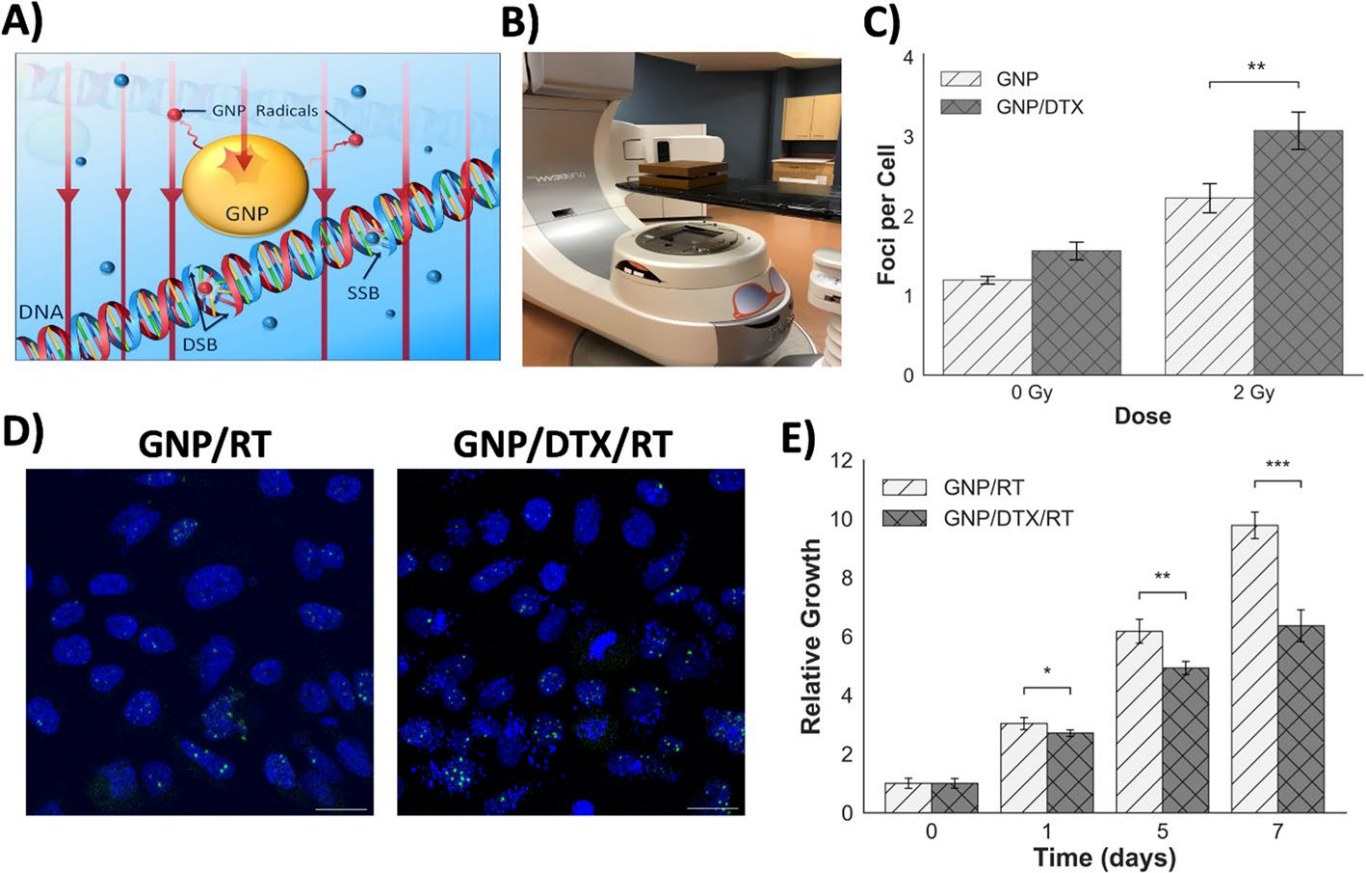
Efficacy analysis of GNP/DTX/RT treatment strategy in vitro. **A** Schematic diagram displaying the mechanism of radiosensitization due to GNPs. GNPs enhance the production of DNA damaging free radicals. **B** Setup used to irradiate cell samples. **C** Quantification of DNA DSBs present in cells 24 h post-irradiation. **D** Confocal images of cells irradiated with a dose of 2 Gy. Nuclei are stained in blue, and 53BP1 DNA repair protein are stained in green. **E** Comparison in the reduction of growth in cells treated with GNPs vs GNP/DTX post-irradiation with a dose of 5 Gy. * indicates 0.01 < *p* < 0.05, ** indicates 0.001 < *p* < 0.01, and *** indicates *p* < 0.001. Scale bar is 20 µm

Our next goal was to evaluate the radiosensitization properties of both GNPs and DTX in PC-3 cells. As discussed previously, GNPs have been pursued as a radiosensitizer due to their higher atomic number (Z_Au_ = 79) (Hainfeld et al. 2004; Butterworth et al. 2013). GNPs enhance radiation damage by producing a shower of secondary electrons when exposed to radiation. These secondary electrons then interact with water and produce free radicals that could damage DNA, as illustrated in Fig. 4A (Zheng and Sanche 2013; Carter et al. 2007). This results in highly localized DNA damaging species, which is essential since locally increasing the dose delivered to tumors can improve treatment outcomes. Recent studies demonstrated that GNP-mediated radiosensitization can be achieved through active cell targeting at ~ 1–5 µg/g concentration of gold within tumors (Wolfe et al. 2015), which is over a thousand-fold lower than that previously thought to be necessary for radiosensitization. For our study, we used 7.5 µg/mL concentration in vitro, such that it translates into our in vivo work. Cells were also treated with DTX at a concentration of 2.72 nM. By arresting cells in the G2/M phase of the cell cycle, DTX further increases the radiosensitivity of cells. Therefore, by incorporating DTX to our GNP/RT treatment strategy, we believe the efficacy of RT could be augmented.

To analyze the efficacy of the treatment strategy, we probed both DNA damage and cell proliferation. A clinical linear accelerator with 6 MV energy was used in our experiment to simulate clinical conditions, and cells were treated with GNPs and DTX 24 h prior to radiation. In the case of DNA damage, DNA double strand breaks (DSBs) were probed, since they are the most lethal to cells. 24 h after a clinically relevant dose of 2 Gy was administered to cell cultures, the DNA damage was assessed by mapping the DNA repair protein 53BP1 (Fig. 4D). A dose of 2 Gy was chosen for this assay to avoid saturation of DNA DSB foci. According to our results, as illustrated in Fig. 4C, there was a significant enhancement in the DNA DSBs of 38% for cells treated with the triple combination of GNP/DTX/RT vs cells treated with GNP/RT. Thus, the results show that the incorporation of DTX amplified the radiotherapeutic effects to cells. This was also supported by our proliferation assay results. For this assay, a RT dose of 5 Gy was delivered to cells, since we used a similar dose for our in vivo work. The addition of DTX to GNP/RT produced a remarkable reduction in cell growth, as shown in Fig. 4E. These radiation results are promising as they display that DTX further increases the radiosensitivity of cells when combined with GNPs (refer to Additional file 1: Sections S5–S6 for control and DTX data). As shown by our uptake analysis, the presence of DTX also increased the accumulation of GNPs in cells. This is crucial, since the radiation dose enhancement properties due to GNPs are dependent on their localization within cells. GNPs closer to the nucleus, if not within the nucleus, are expected to produce optimum radiation damage during RT (Yuting et al. 2015; Yang et al. 2014a). With the addition DTX, cells are blocked in the G2/M phase of the cell cycle and GNPs become trapped around nucleus as a result of MT stabilization. Therefore, there exists a synergistic relationship between GNPs and DTX, and both factors could contribute to a better therapeutic outcome of this triple combination of GNP/DTX/RT.

### In vivo analysis of GNP/DTX/RT treatment strategy

The in vitro results of the triple combination of GNP/DTX/RT were very promising. It is generally recognized that in vitro results cannot be extrapolated directly to in vivo settings (Hill and Robert 2008). Several extra factors must be considered to extend in vitro work to in vivo studies (Bae and Park 2011). As a result, the outcome of in vitro nanomedicine research is sometimes overstated, and in vivo outcomes often fall short of expectations (Chouikrat et al. 2012; Alkilany and Murphy 2010). Hence, we optimized the surface functionality and size of our GNPs using both two-dimensional (2D) and 3D tissue-like models before using them in in vivo experiments. In this study, we have chosen GNPs of diameter 11 nm functionalized with both PEG and RGD peptide as previous in vitro and in vivo studies support this GNP complex (Bromma et al. 2022; Yang et al. 2014b). In this study, we have chosen GNPs of diameter 11 nm functionalized with both PEG and RGD peptide as previous in vitro and in vivo studies support this GNP complex. The key attributes of our approach include intravenous administration of thousand-fold lower concentrations (mg/Kg instead of mg/g), clinically relevant radiation beam energies (megavoltage (MV) radiation using a clinical linear accelerator), a single injection before RT treatment, and the RT dose delivered a day after intravenous nanoparticle and DTX administration.

**Fig. 5:**
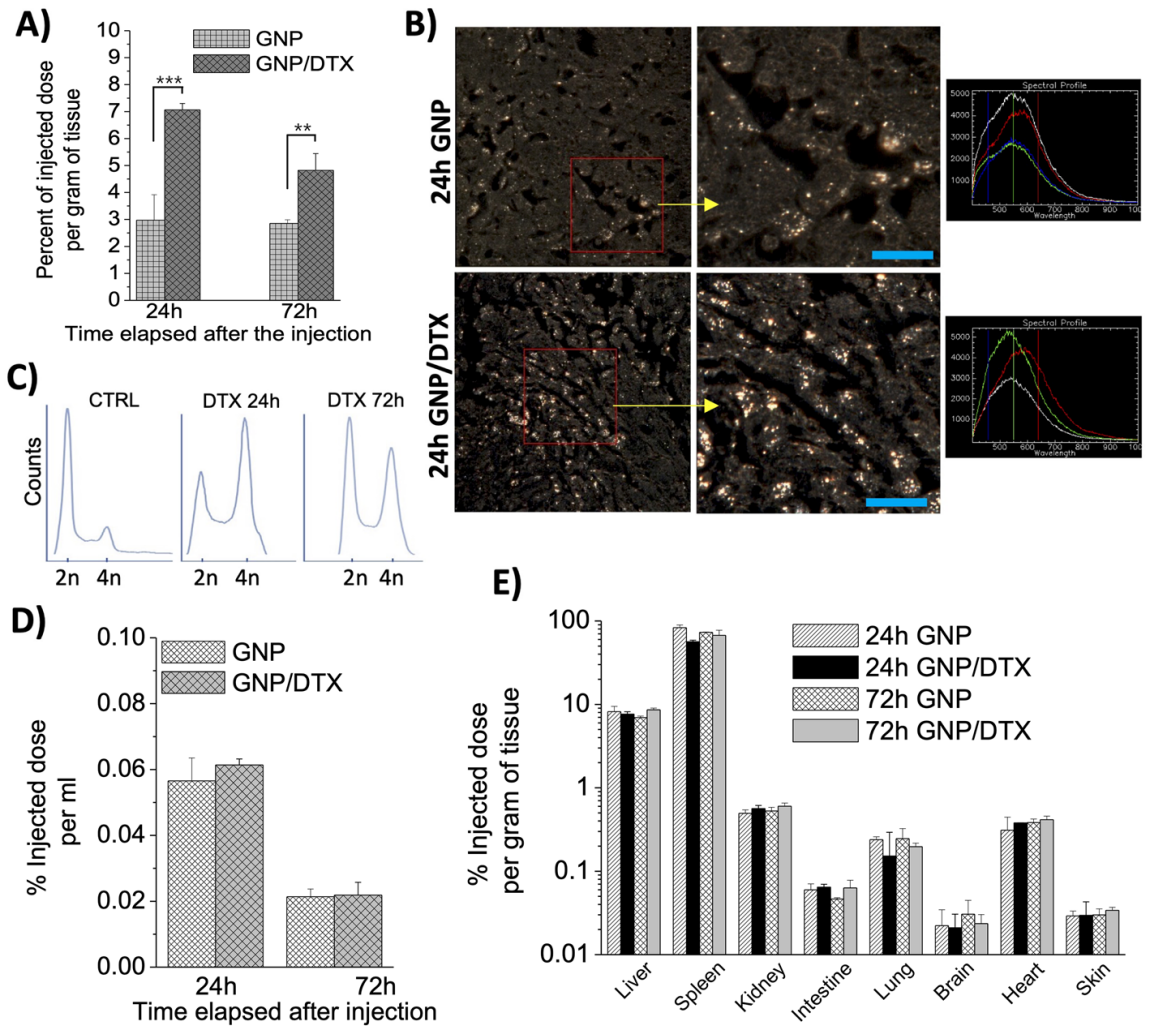
Biodistribution analysis of GNPs. **A** Percentage of total injected dose of GNP content per gram of mouse tumor tissue in control (no DTX) tissue and DTX treated tissue. **B** Hyperspectral images comparing the GNP accumulation in control tumor tissue vs DTX treated tumor tissue 24 h post-treatment. Spectra are collected from GNP clusters. **C** Cell cycle analysis of tumor tissue samples treated with and without DTX. **D**, **E** Quantitative comparison of GNP content in blood and various organs 24 h and 72 h after mice were treated with GNPs and/or DTX. ** indicates 0.001 < *p* < 0.01, and *** indicates *p* < 0.001. Scale bar is 40 µm

We first looked at the accumulation of GNPs a day after intravenous nanoparticle administration. Once tumors reached 7–8 mm in longitudinal diameter, intravenous injections of GNPs and DTX (free drug) were administered to mice to conduct the biodistribution study. A GNP dose of 2 mg/kg (mouse) and a DTX dose of 6 mg/kg (mouse) were used. The injected dose is well below the reported LD50 for GNPs (Hainfeld et al. 2004). To roughly match a weekly regimen of DTX in humans (~ 20 mg/m^2^), a dose of 6 mg/kg was chosen (Nawrocki et al. 2004; Bradshaw-Pierce et al. 2008). The accumulation (or retention) of GNPs and the cell cycle phase of the tumor cell population were tested 1 and 3 days after the initial injection, and at the end of each timepoint, tumor tissue was used for imaging (using dark-field microscopy), cell cycle (Flow Cytometry) analysis, and GNP quantification (using ICP-MS). We also analyzed the GNP content in the blood, liver, spleen, kidneys, intestine, brain, heart, and skin. These analyses allowed us to determine the optimal time for RT delivery based on the GNP accumulation in the tumor as well as the synchronization of cells in the G2/M phase. As shown in Fig. 5A, there was more than a 100% increase in GNP accumulation within the tumor in the presence of DTX 24 h post-injection. This was further supported by qualitative dark-field images as a clear contrast in the accumulation of gold in the control tumor cells vs the DTX treated cells is present (Fig. 5B). Interestingly, these results exhibit a greater relative increase in the uptake of GNPs in the tumor in comparison with the in vitro results. Although inconclusive, this insinuates the concentration of DTX experienced by tumor cells was higher than the 2.72 nM used for the in vitro analysis, which could correspond to an increase in the radiosensitivity of the tumor in comparison with the monolayer cell cultures. Furthermore, as explained before in our in vitro experimental results section, we believe DTX plays a major role in trapping GNPs within tumor cells for a greater period of time. Therefore, we measured the phase of tumor cell population with the treatment of DTX. As shown in Fig. 5C, there was a larger cell population in G2/M phase at the 24 h timepoint. It was also notable that cell population stayed in G2/M phase even after 72 h. This could be one of the reasons that DTX is given weekly to patients in the clinic. It is also important to note that the addition of DTX did not significantly increase the accumulation of GNPs in blood as well as the organs (Fig. 5D, E). Thus, the addition of DTX facilitates an increase in the uptake and retention of GNPs within the tumor while having an insignificant impact on organs.

Our in vivo data in Fig. 5C displays the cell cycle arrest in G2/M phase in alignment with maximum GNP accumulation at the 24 h timepoint, signaling maximum radiosensitivity of tumor cells. At this optimal timepoint, a single RT dose of 5 Gy was delivered using a 6 MV linear accelerator. The development of more advanced radiotherapy delivery techniques such as intensity modulated radiotherapy and volumetric modulated arc therapy has enabled a transition towards stereotactic body radiotherapy (SBRT) for cancer patients, where hypofractionated doses such as 5 Gy are applied. Recent results from PACE-B and HYPO-RT-PC clinical trials support the clinical feasibility of ultra hypofractionation for prostate cancer patients (Tree et al. 2022; Fransson et al. 2021). Therefore, we chose 5 Gy as the radiation dose for our studies. Furthermore, using a dose of 5 Gy ensures the mice will survive the radiation, allowing for the study of later endpoints (Biedermann et al. 1991). Tumor size and body weight were measured three times a week and mice were euthanized at 20% body weight loss, excessive clinical observations, or the experimental endpoint of the tumor volume reaching 1000 mm^3^. The treatment arms for the single RT dose delivery were control (PBS), GNPs, DTX, and GNP/DTX. We also had non-radiated arm with the same conditions for comparison. As mentioned before, a lower dose of DTX was used to minimize side effects. For example, in phase I/II trials, the weekly DTX dose was reduced from 25–40 mg/m^2^ to 20 mg/m^2^ for prostate cancer and to 10 mg/m^2^ in locally advanced head and neck cancer (Kumar 2003; Kumar et al. 2003; Karasawa, et al. 2006). Therefore, we scaled back the DTX dose to 20 mg/m^2^ in our prostate model, which is roughly equivalent to 6 mg/kg in a mouse model. Furthermore, this dose of DTX is not expected to significantly impact the tumor growth delay when used alone (Mosallaei et al. 2013; Nishizaki et al. 2001). Instead, its therapeutic effect is to increase the radiosensitivity of tumors.

**Fig. 6:**
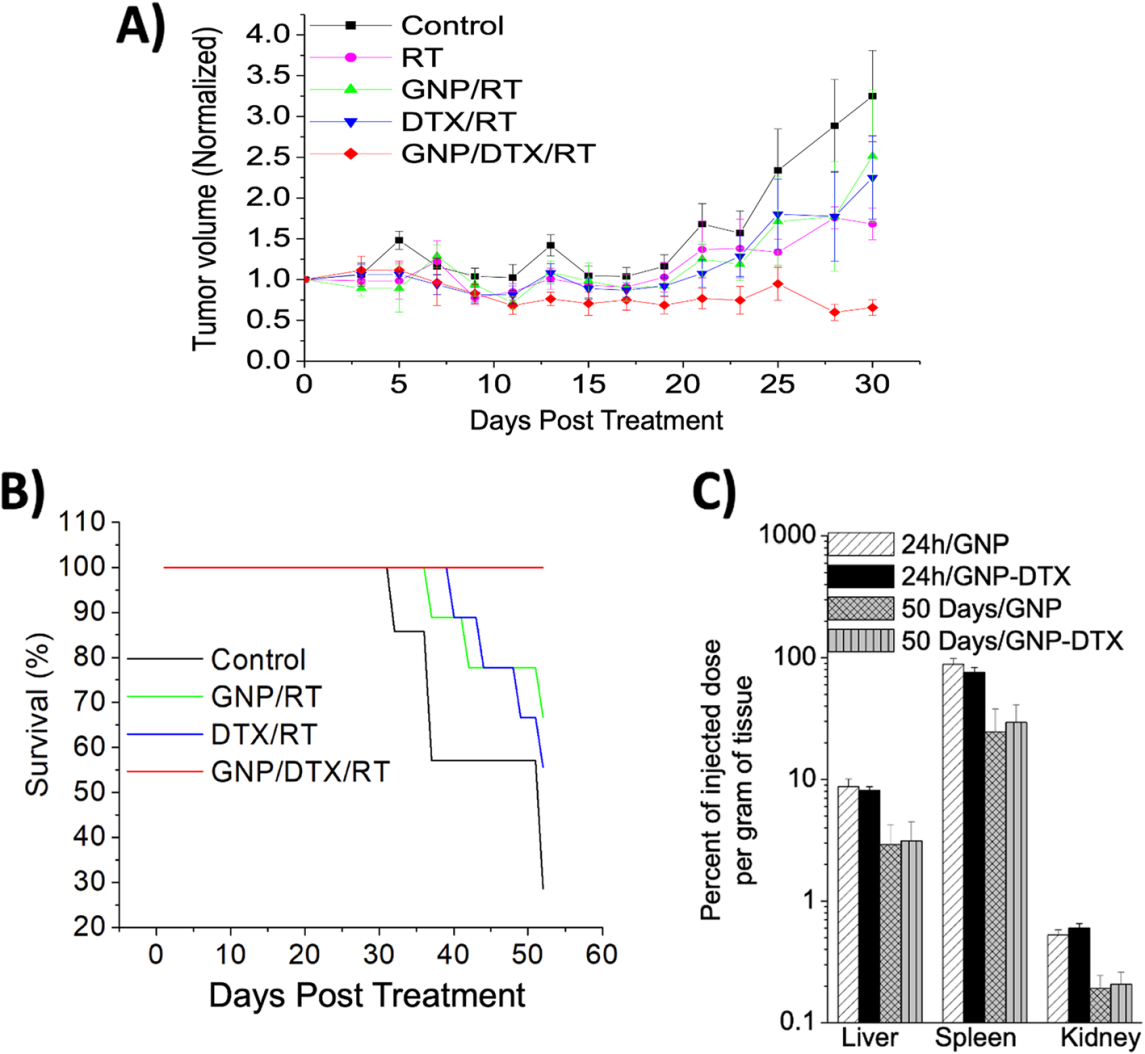
Evaluation of GNP/DTX/RT treatment in vivo. **A** Measurements of normalized mice tumor volume size post-treatment to display the reduction of growth of mice treated with RT, GNP/RT, DTX/RT, and GNP/DTX/RT. Results are presented as a normalized average tumor volume of at least five mice (mean ± standard deviation). **B** Variation in the mouse survival fraction for mice that were treated with RT, GNP/RT, DTX/RT, and GNP/DTX/RT. **C** Presence of GNPs in liver, spleen, and kidney of control mice (no DTX) and DTX treated mice 24 h and 50-day post-treatment

Figure 6A exhibits the results in the variation of tumor volume after mice received treatment. There was a significant difference in tumor growth delay with the triple combination of GNP/DTX/RT compared to GNP/RT after 30 days (*p* = 0.02). Our results also displayed significant tumor growth delay for the GNP/DTX/RT condition up to 40-day post-treatment (Additional file 1: Figure S10). Furthermore, it was found that the survival of mice was greatly improved with the treatment of GNP/DTX/RT, corresponding with the tumor growth delay data (Fig. 6B). This suggests that the treatment strategy is advantageous in vivo as well as in vitro, and thus, GNPs combined with DTX can further increase the radiosensitivity of tumor cells. Therefore, if translated to clinical applications, the synergistic relationship between the triple combination of GNP/DTX/RT could have vast implications on the curative results and could potentially increase the overall survival rate. Pioneering study by Hainfeld et al. was first shown to increase radiocurability of tumors, where mice were given a 30 Gy dose with 250 kVp X-rays immediately (2 min) after intravenous administration of 1.9 nm diameter GNPs (Hainfeld et al. 2004). However, the concentration of GNPs was 2.7 g Au/kg body weight, which is not clinically feasible. We believe such high concentrations were needed, since GNPs were not localized intracellularly. In contrast, whenever gold was internalized, radiosensitization was achievable at concentrations as low as 10^–6^ mg/g (Zhang et al. 2008; Chang et al. 2008). In this study, we used a gold dose of 2 mg Au/kg, which is thousand times less and makes it more clinically feasible. The GNPs used in our study were also tailored in size and surface properties for better penetration within tumor and intracellular uptake at individual cell level. In addition, the use of MV energies vs kV allows penetration for deep-set tumors. Along with the lower dose of DTX as used in this study, our treatment strategy could perhaps also enable the reduction in some of the normal toxicities associated with chemotherapeutic drugs while also improving tumor control.

One of the concerns raised amongst the research community is the accumulation and retention of GNPs in organs, such as the liver, kidney, and spleen, as some have concerns about the possible toxicity GNPs can induce. Thus, we monitored mice over a 50-day period (Fig. 6C). As shown, the accumulation of GNPs in the organs decreased, but there was still a considerable amount retained. Furthermore, much of the injected dose of GNPs accumulated in the organs of the mice in comparison with the tumor (Fig. 5E). Reducing this differential in accumulation of GNPs in tumor sites relative to normal tissue could facilitate an easier transition into clinical settings as it could be possibly met with less resistance from clinicians. One strategy that could be implemented to achieve this is through intratumoral injection, a strategy employed by Nanobiotix, a biotech company that has tested high-Z radiosensitizing nanoparticles in clinical trials. NBTXR3, functionalized hafnium oxide nanoparticles, are radioenhancers produced by Nanobiotix, and they have shown effective anticancer effects in clinical trials (Bonvalot, et al. 2019). Similar to GNPs, hafnium oxide nanoparticles locally enhance the dose due to an increase in ionizing interactions. NBTXR3 has also received CE Mark approval for the treatment of locally advanced soft tissue carcinoma via intratumoral injection, displaying the benefits of using nanoparticles as radiosensitizers to improve clinical outcomes for cancer patients (Anselmo and Mitragotri 2019). By implementing intratumoral injection of GNPs into our treatment strategy, it could possibly assist the transition of our strategy into clinical settings, as well as provide a more efficient therapeutic pathway.

Although our in vitro and in vivo studies were carried out using a standard prostate cancer cell line, PC-3, in a real tumor microenvironment (TME), there are many other cell types present in addition to tumor cells. Among other cell types, cancer associated fibroblasts (CAFs) within the TME play a significant role in tumor growth (Albrengues et al. 2015). They promote the proliferation of tumors through the release of cytokines and chemokines, such as vascular endothelial growth factors, and through extracellular matrix (ECM) remodelling involving matrix metalloproteinases (Alkasalias et al. 2018; Stetler-Stevenson et al. 1993). They also play a significant role in tumor metastasis and chemoresistance (Karagiannis et al. 2012; Dilworth et al. 2018). For this reason, it is also important to consider the efficacy of a treatment strategy on CAFs. A previous study found that the DTX substantially increased the uptake of GNPs in CAFs (Alhussan et al. 2021). This increase in the presence of GNPs could lead to reduced activity of CAFs with radiation treatment. A recent co-culture study of tumor cells and CAFs of pancreatic origin showed reduced activity of CAFs with the treatment of GNP/RT (Alhussan et al. 2022). This suggests that the addition of DTX would further improve the efficacy of GNP/RT on CAFs, and, therefore, be effective when applied to a proper TME. Thus, we believe our novel approach of GNP/DTX/RT could make a significant improvement in the treatment outcome of cancer patients.

## Conclusion

Radiotherapy is a crucial component in many cancer treatment regimens. By preferentially increasing the effect of the dose delivered to tumors, the curative results can be further improved. This necessitates the need for a novel approach that can locally increase the damage to tumors while still maintaining low levels of normal tissue toxicity. Our proposed method of combining GNPs and clinically used DTX can achieve this by increasing the sensitivity of cancer cells to RT treatment. GNPs have already been shown to selectively increase the RT dose delivered to tumors. With the addition of DTX, tumors can be further sensitized to radiation. DTX arrests cells in the most radiosensitive phase and increases the accumulation of GNPs within tumors, creating a synergistic relationship between GNPs, DTX, and RT. In our study, we used clinically feasible concentrations for both GNP and DTX to test the efficacy of the treatment strategy. The radiation treatment was delivered at the optimal timepoint of 24 h post-injection, which was determined by the results of the biodistribution and cell cycle analysis. Furthermore, our radiation results displayed that the triple combination of GNP/DTX/RT had significant impact on reducing the growth of tumor cells, both in vitro and in vivo. These promising results suggest that this modern approach to gain more local control of the disease could vastly improve treatment outcomes and the quality of life of cancer patients.

### Supplementary Information

**Additional file 1: Figure S1**. GNP characterization. (**A**) UV Visible spectra for GNP, GNP–PEG and GNP–PEG–RGD. (**B) **DLS measurements of GNP–PEG–RGD complex 2 months after functionalization with ligands. Water, PBS and DMEM cell culture media were used as solvents. (**C**) Summary of UV–Vis, DLS, and zeta potential data collected at each step of the functionalization process. **Figure S2**. Cell division and GNP distribution in cells treated with or without docetaxel. First column displays a cell attempting to undergo cell division. Microtubules are stained in green, and GNPs are stained in red. Scale bar = 20 µm. **Figure S3.** Hyperspectral images of cells 24 h post-treatment using a dark-field microscope. Cells were either left untreated (control) or treated with either GNP or GNP/DTX. Spectra are taken from GNP clusters or the cell body. Scale bar = 20 µm. **Figure S4**. Cell cycle analysis of PC-3 cells that were left untreated (control), or treated with DTX, 24 h and 72 h post-dosing. **Figure S5. **Comparison of cell proliferation for control (CTRL) cells (no treatment) vs GNP treated cells (GNP) in the absence of radiation. **Figure S6**. *In vitro* radiation assay results. (**A**) Confocal images of cells 24 h after being irradiated with a dose of 2 Gy. Nuclei are stained in blue, 53BP1 DNA damage repair protein are stained in green. (**B**) Quantification of DNA double-strand breaks in control cells (no DTX) or DTX treated cells, 24 h after a dose of 2 Gy was administered. (**C**) Comparison in the reduction of growth in control cells (no DTX) or cells treated with DTX that were irradiated at a dose of 5 Gy.** Figure S7**. Hyperspectral images of *in vivo *tumor tissue samples 24 h and 72 h after mice were treated with GNP or GNP/DTX. Spectra are taken from GNP clusters. Scale bar = 40 µm.** Figure S8**. Hyperspectral images of spleen, kidney, liver, and lung samples from mice treated with GNP/DTX. Scale bar = 40 µm. **Figure S9. **Qualitative comparison of GNP+PEG vs GNP+PEG/RGD accumulation in PC-3 cells. Scale bar = 20 µm. **Figure S10. **Measurements of mice tumor volume size post-treatment to display the reduction of growth of mice treated with RT, GNP/RT, DTX/RT, and GNP/DTX/RT. Results are presented as an average tumor volume of at least five mice (mean ± standard deviation). For each condition, data are displayed up until the first mice was sacrificed in their respective treatment group.

## Data Availability

Data sets generated and/or analyzed during the current study are available from the corresponding author on reasonable request.
