# Supplementary material for: Dual enhancement in the radiosensitivity of prostate cancer through nanoparticles and chemotherapeutics
Source: Cancer Nanotechnol. 2023 Sep 29;14(1):75. doi: 10.1186/s12645-023-00228-0 (PMC10539438; doi:10.1186/s12645-023-00228-0)
Supplement: Supplementary file 1 — Additional file 1: Figure S1. GNP characterization. (A) UV Visible spectra for GNP, GNP–PEG and GNP–PEG–RGD. (B) DLS measurements of GNP–PEG–RGD complex 2 months after functionalization with ligands. Water, PBS and DMEM cell culture media were used as solvents. (C) Summary of UV–Vis, DLS, and zeta potential data collected at each step of the functionalization process. Figure S2. Cell division and GNP distribution in cells treated with or without docetaxel. First column displays a cell attempting to undergo cell division. Microtubules are stained in green, and GNPs are stained in red. Scale bar = 20 µm. Figure S3. Hyperspectral images of cells 24 h post-treatment using a dark-field microscope. Cells were either left untreated (control) or treated with either GNP or GNP/DTX. Spectra are taken from GNP clusters or the cell body. Scale bar = 20 µm. Figure S4. Cell cycle analysis of PC-3 cells that were left untreated (control), or treated with DTX, 24 h and 72 h post-dosing. Figure S5. Comparison of cell proliferation for control (CTRL) cells (no treatment) vs GNP treated cells (GNP) in the absence of radiation. Figure S6. In vitro radiation assay results. (A) Confocal images of cells 24 h after being irradiated with a dose of 2 Gy. Nuclei are stained in blue, 53BP1 DNA damage repair protein are stained in green. (B) Quantification of DNA double-strand breaks in control cells (no DTX) or DTX treated cells, 24 h after a dose of 2 Gy was administered. (C) Comparison in the reduction of growth in control cells (no DTX) or cells treated with DTX that were irradiated at a dose of 5 Gy. Figure S7. Hyperspectral images of in vivo tumor tissue samples 24 h and 72 h after mice were treated with GNP or GNP/DTX. Spectra are taken from GNP clusters. Scale bar = 40 µm. Figure S8. Hyperspectral images of spleen, kidney, liver, and lung samples from mice treated with GNP/DTX. Scale bar = 40 µm. Figure S9. Qualitative comparison of GNP+PEG vs GNP+PEG/RGD accumulation in PC-3 cells. Sca [file 12645_2023_228_MOESM1_ESM.docx]

**Additional file**

**Dual enhancement in the radiosensitivity of prostate cancer through nanotechnology and chemotherapeutics**

**Additional section S1:**


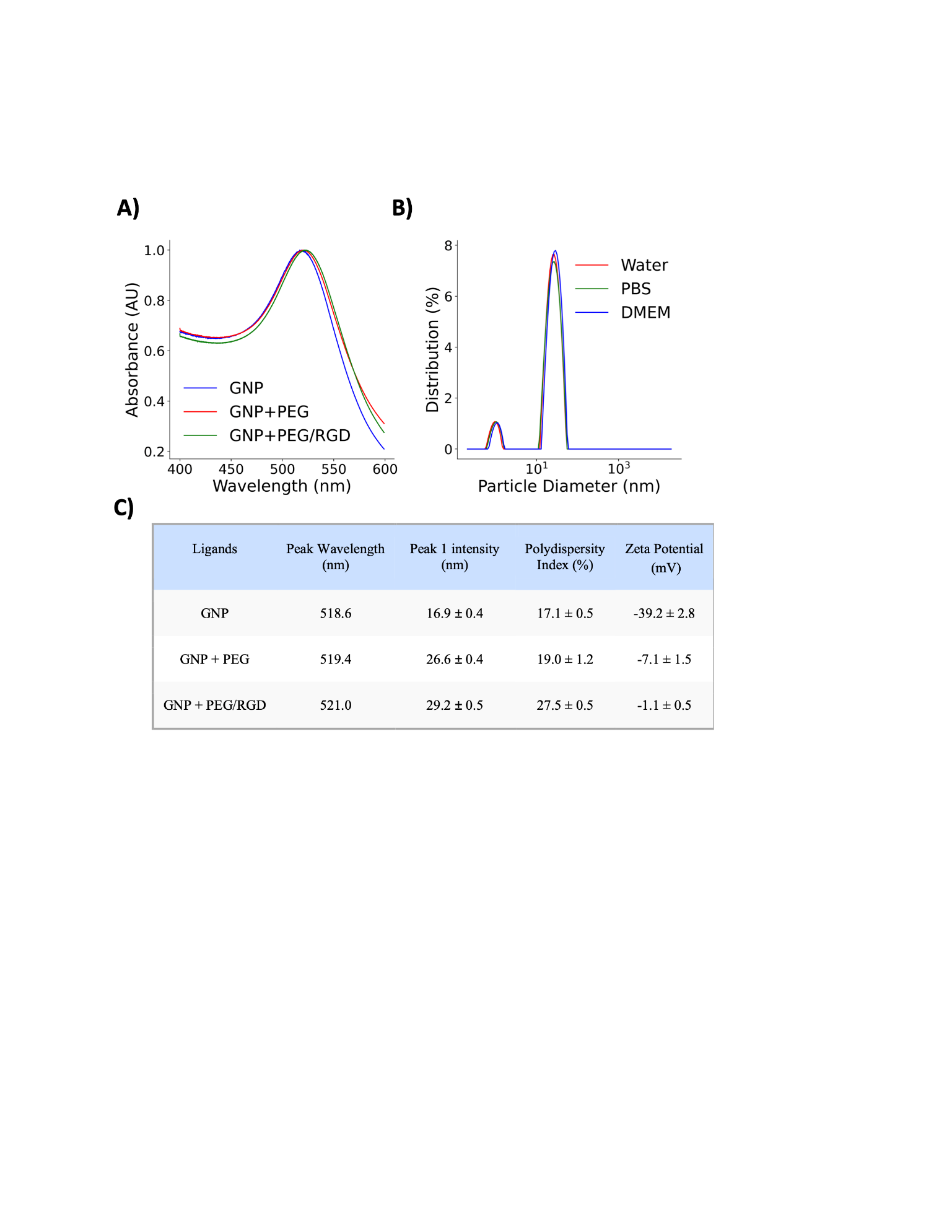


**Figure S1**. GNP characterization. (**A**) UV Visible spectra for GNP, GNP-PEG and GNP-PEG-RGD. (**B)** DLS measurements of GNP-PEG-RGD complex 2 months after functionalization with ligands. Water, PBS and DMEM cell culture media were used as solvents. (**C**) Summary of UV-VIS, DLS, and zeta potential data collected at each step of the functionalization process.

**
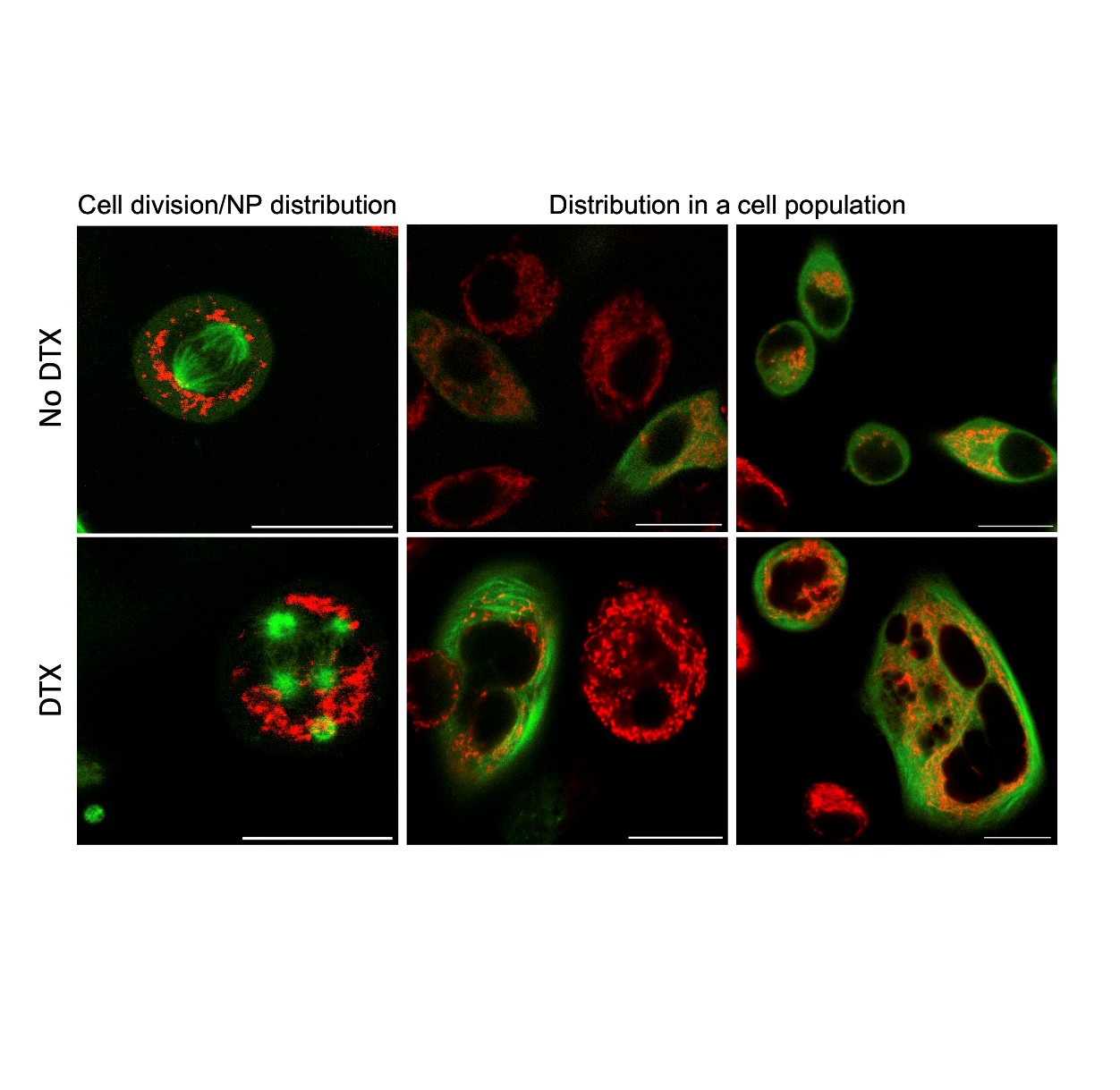
Additional section S2:**

**Figure S2**. Cell division and GNP distribution in cells treated with or without docetaxel. First column displays a cell attempting to undergo cell division. Microtubules are stained in green, and GNPs are stained in red. Scale bar = 20 µm.

**Additional section S3:**

**
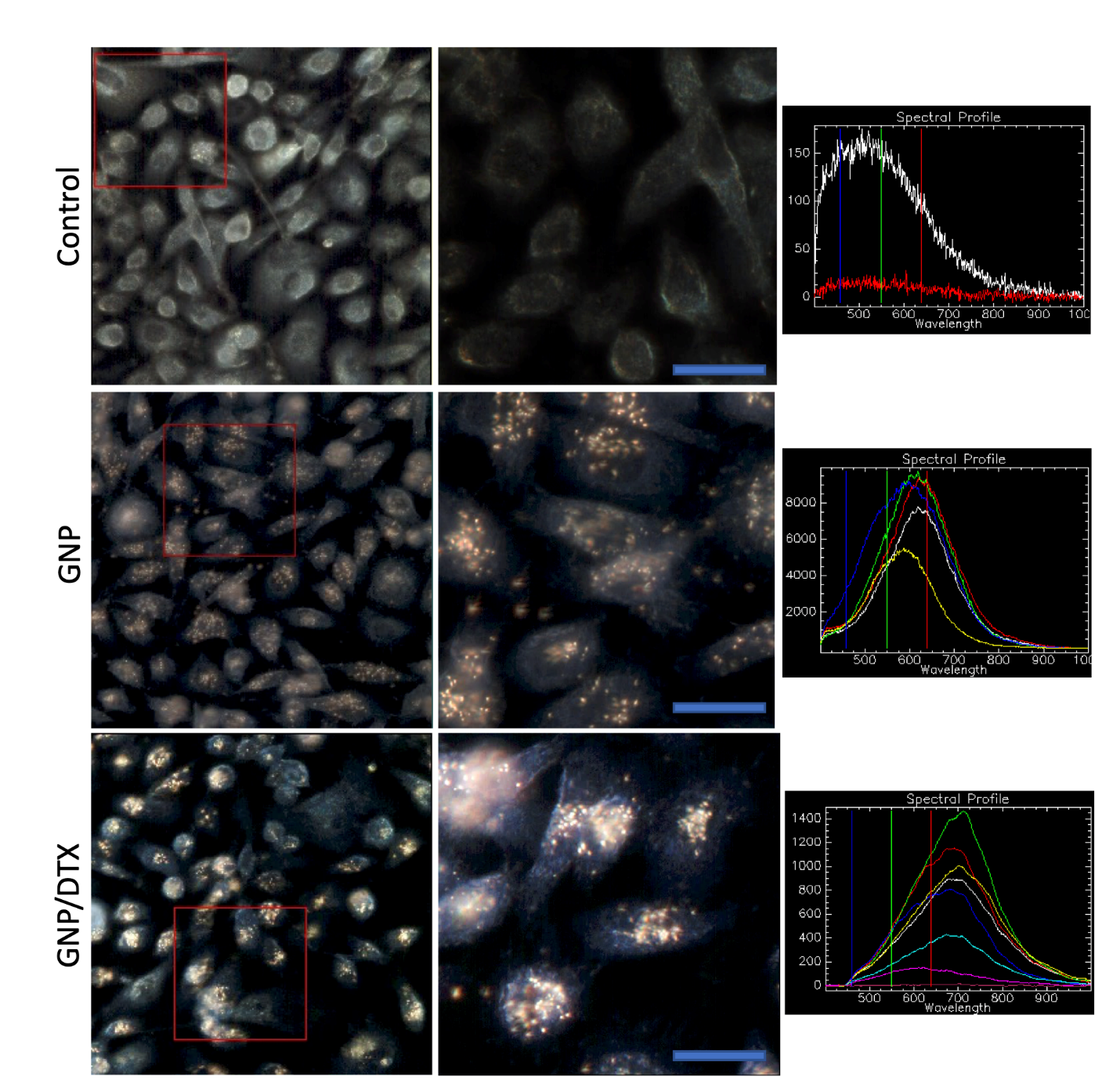
**

**Figure S3.** Hyperspectral images of cells 24 h post treatment using a dark-field microscope. Cells were either left untreated (control) or treated with either GNP or GNP/DTX. Spectra are taken from GNP clusters or the cell body. Scale bar = 20 µm.

**Additional section S4:**

**
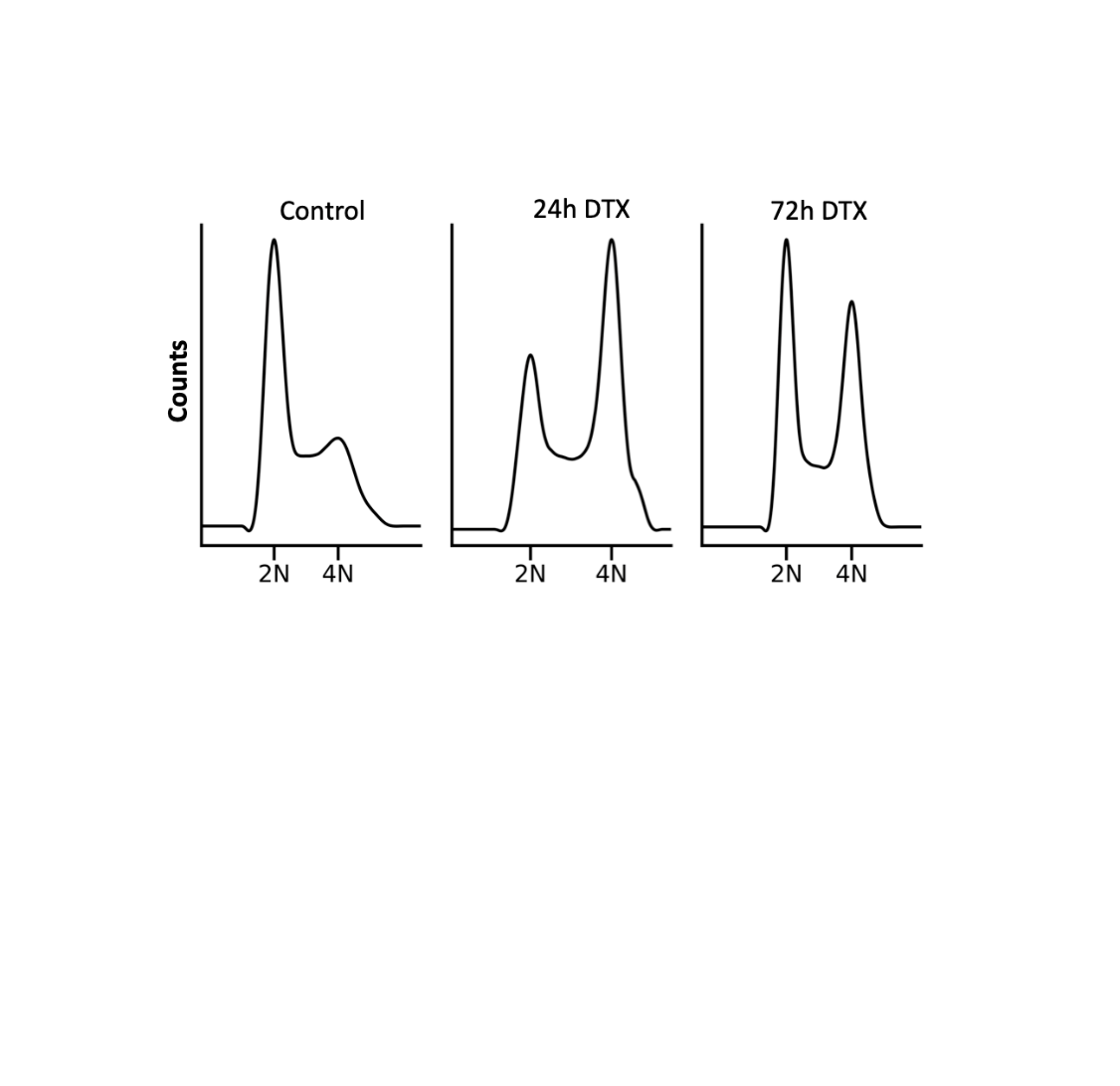
**

**Figure S4**. Cell cycle analysis of PC-3 cells that were left untreated (control), or treated with DTX, 24 h and 72 h post dosing.

**Additional section S5:**

**
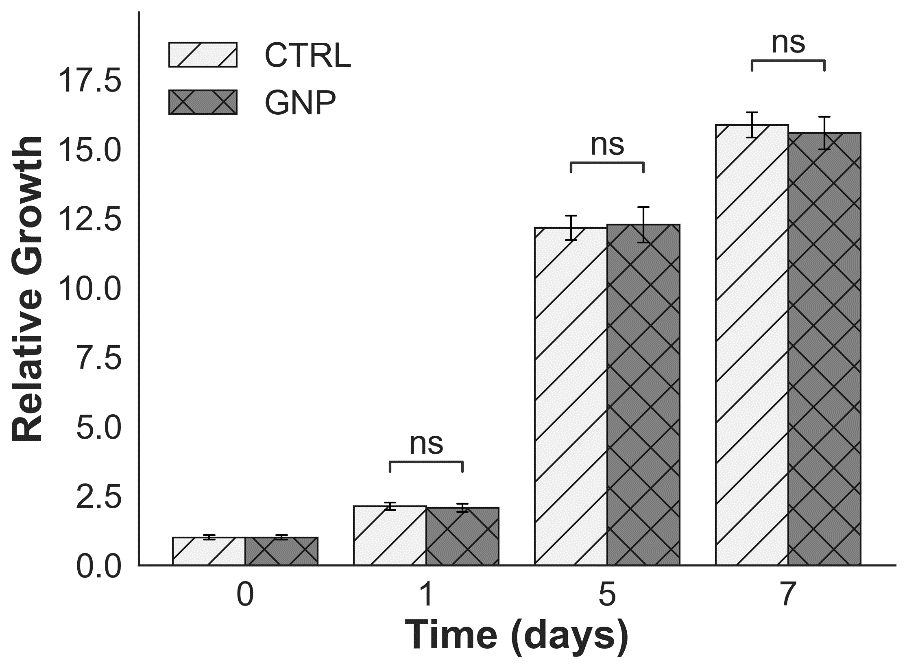
**

**Figure S5:** Comparison of cell proliferation for control (CTRL) cells (no treatment) versus GNP treated cells (GNP) in the absence of radiation.

**Additional section S6:**

**
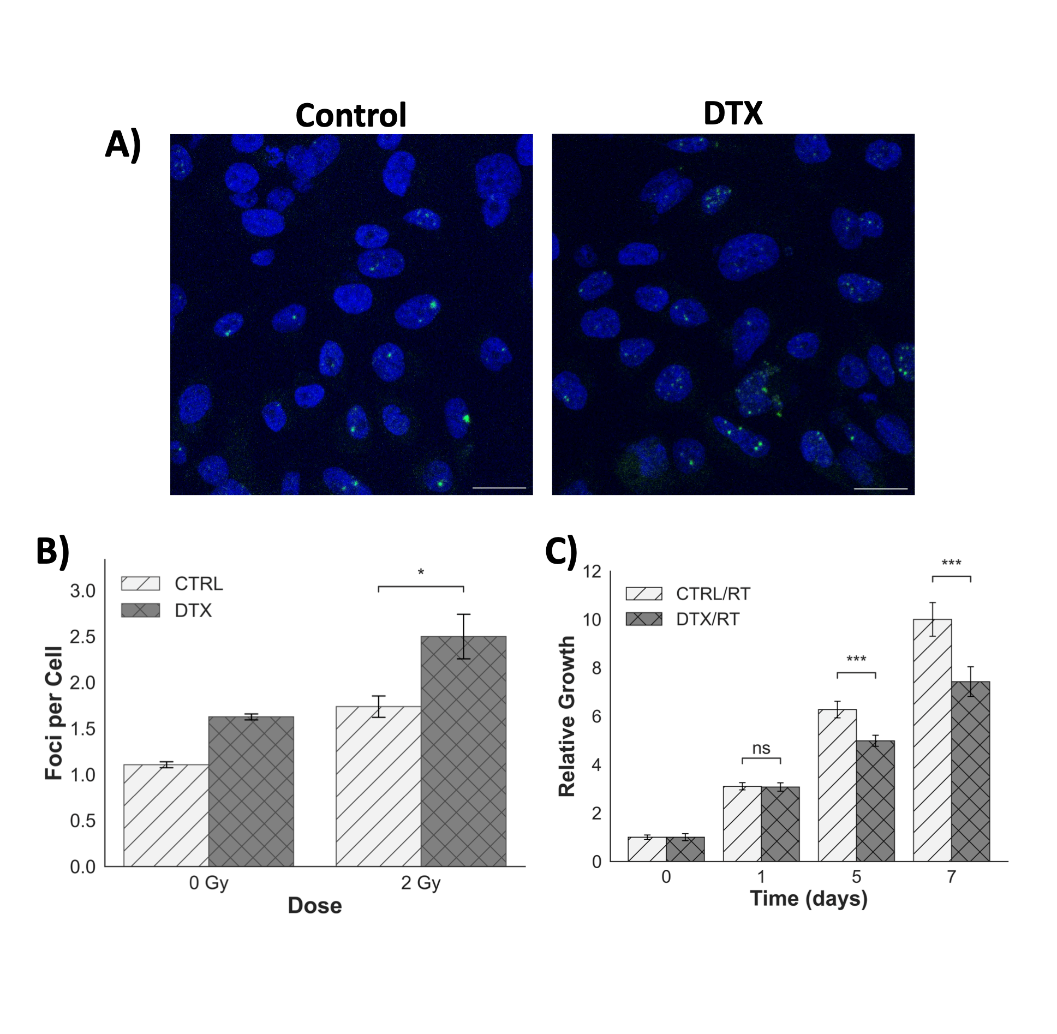
**

**Figure S6**. *In vitro* radiation assay results. (**A**) Confocal images of cells 24 h after being irradiated with a dose of 2 Gy. Nuclei are stained in blue, 53BP1 DNA damage repair protein are stained in green. (**B**) Quantification of DNA double-strand breaks in control cells (no DTX) or DTX treated cells, 24 h after a dose of 2 Gy was administered. (**C**) Comparison in the reduction of growth in control cells (no DTX) or cells treated with DTX that were irradiated at a dose of 5 Gy.

**Additional section S7:**

**
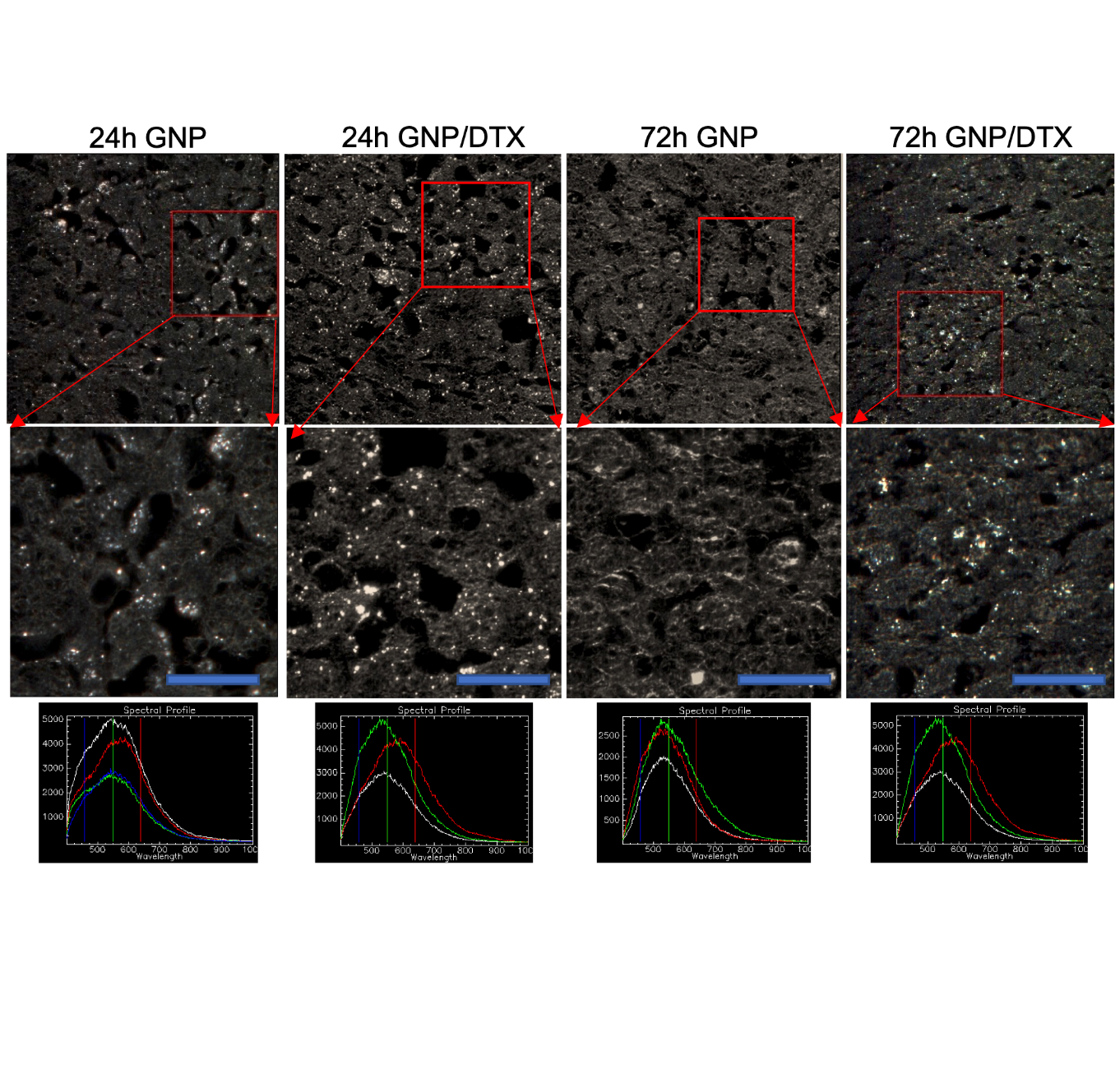
**

**Figure S7**. Hyperspectral images of *in vivo* tumor tissue samples 24 h and 72 h after mice were treated with GNP or GNP/DTX. Spectra are taken from GNP clusters. Scale bar = 40 µm.


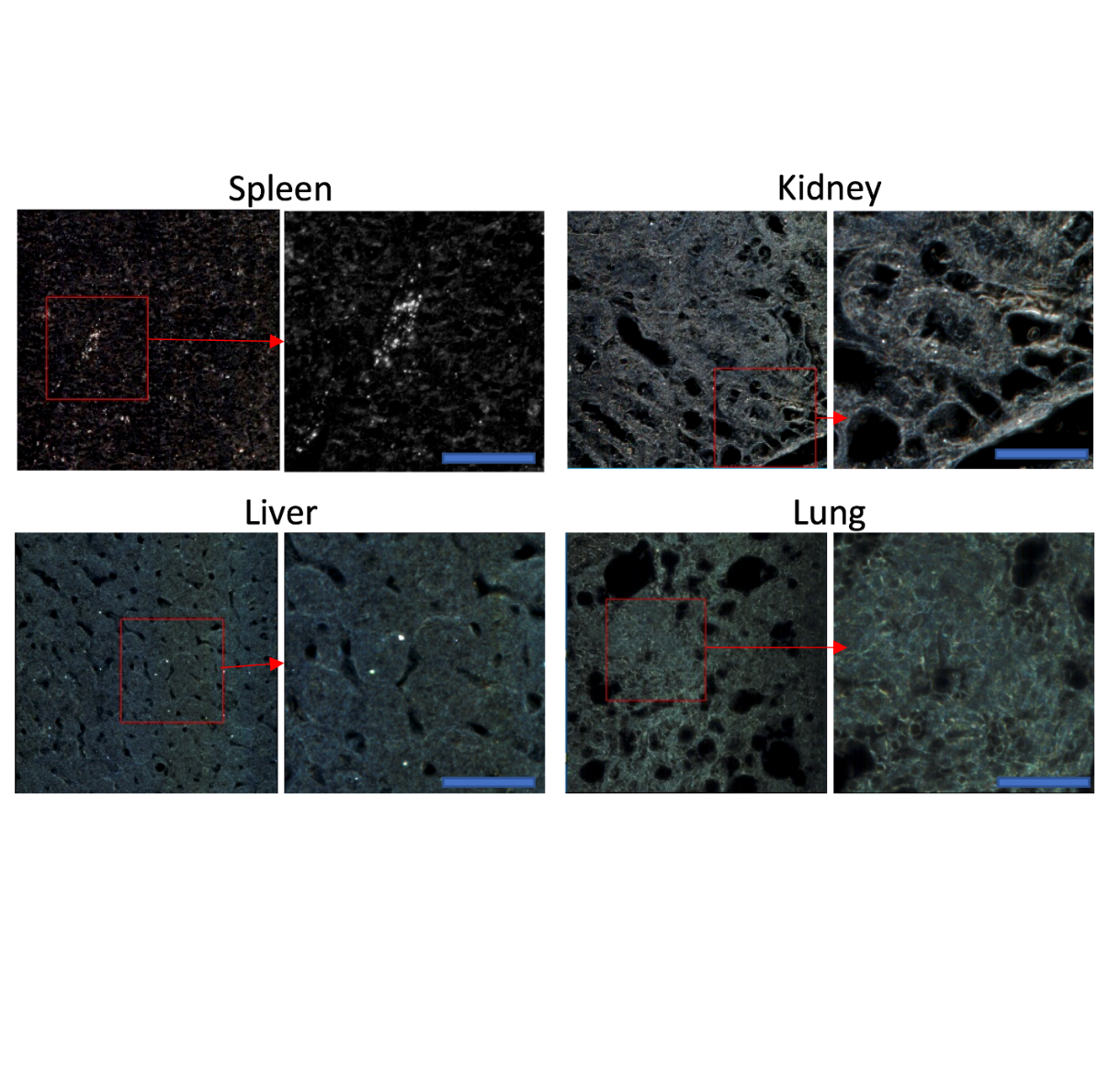
**Additional section S8:**

**Figure S8**. Hyperspectral images of spleen, kidney, liver, and lung samples from mice treated with GNP/DTX. Scale bar = 40 µm.

**Additional section S9:**

**
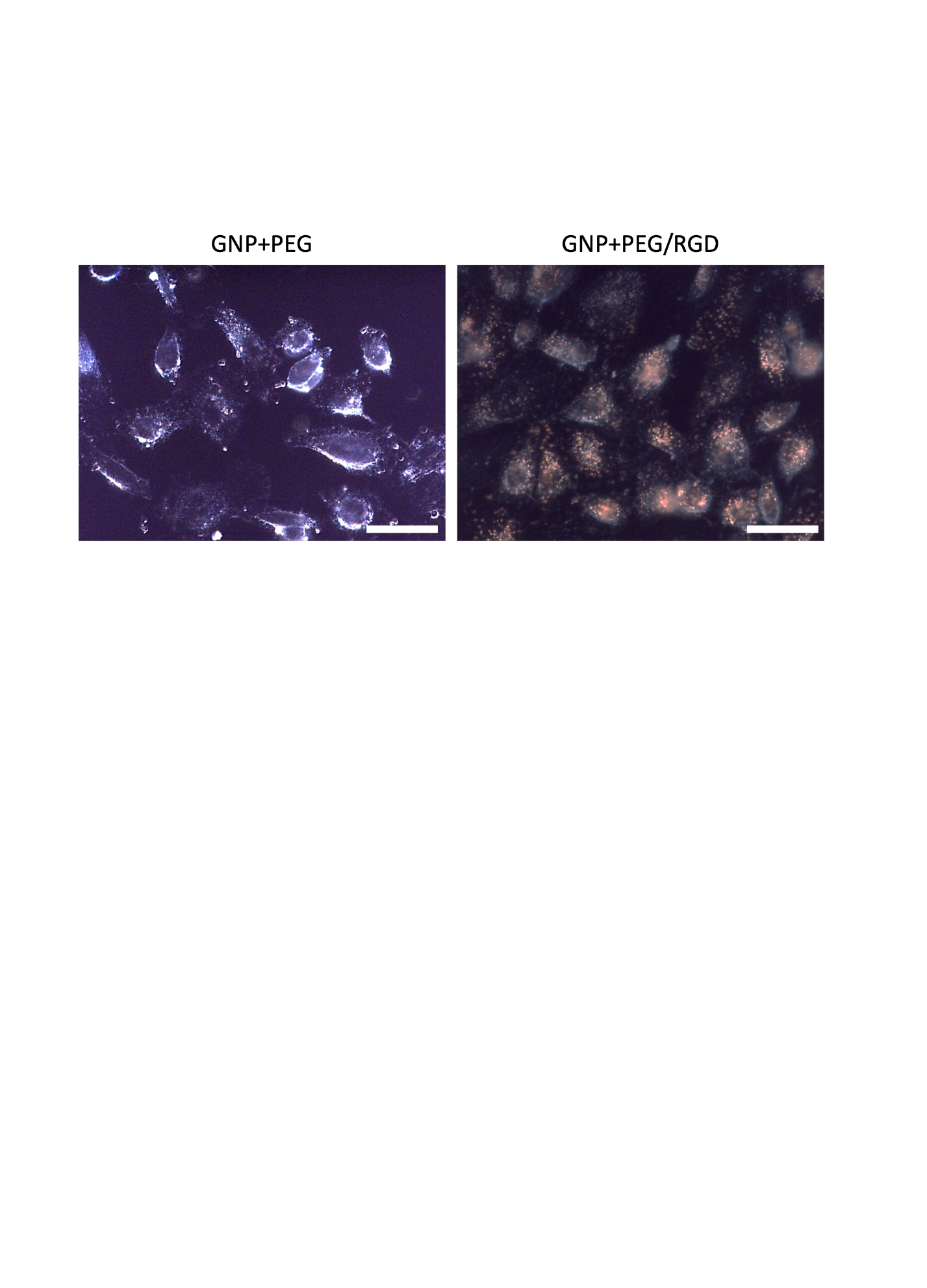
**

**Figure S9:** Qualitative comparison of GNP+PEG vs GNP+PEG/RGD accumulation in PC-3 cells. Scale bar = 20 µm.

**Additional section S10:**

**Figure S10:** Measurements of mice tumor volume size post treatment to display the reduction of growth of mice treated with RT, GNP/RT, DTX/RT, and GNP/DTX/RT. Results are presented as an average tumor volume of at least five mice (mean ± standard deviation). For each condition, data is displayed up until the first mice was sacrificed in their respective treatment group.
